# Functional Diversification, Redundancy, and Epistasis among Paralogs of the *Drosophila melanogaster Obp50a–d* Gene Cluster

**DOI:** 10.1093/molbev/msab004

**Published:** 2021-02-09

**Authors:** Joel A Johnstun, Vijay Shankar, Sneha S Mokashi, Lakshmi T Sunkara, Ugonna E Ihearahu, Roberta L Lyman, Trudy F C Mackay, Robert R H Anholt

**Affiliations:** 1 Department of Biological Sciences, Program in Genetics and W.M. Keck Center for Behavioral Biology, North Carolina State University, Raleigh, NC, USA; 2 Department of Genetics and Biochemistry and Center for Human Genetics, Clemson University, Greenwood, SC, USA; 3 Faculty of Health and Medical Sciences, University of Surrey, Guildford, United Kingdom

**Keywords:** multigene families, functional diversification, transcriptional niche, RNAseq, odorant-binding proteins, evolutionary genetics

## Abstract

Large multigene families, such as the insect odorant-binding proteins (OBPs), are thought to arise through functional diversification after repeated gene duplications. Whereas many OBPs function in chemoreception, members of this family are also expressed in tissues outside chemosensory organs. Paralogs of the *Obp50* gene cluster are expressed in metabolic and male reproductive tissues, but their functions and interrelationships remain unknown. Here, we report the genetic dissection of four members of the *Obp50* cluster, which are in close physical proximity without intervening genes. We used CRISPR technology to excise the entire cluster while introducing a *PhiC31* reintegration site to reinsert constructs in which different combinations of the constituent *Obp* genes were either intact or rendered inactive. We performed whole transcriptome sequencing and assessed sexually dimorphic changes in transcript abundances (transcriptional niches) associated with each gene-edited genotype. Using this approach, we were able to estimate redundancy, additivity, diversification, and epistasis among *Obp50* paralogs. We analyzed the effects of gene editing of this cluster on organismal phenotypes and found a significant skewing of sex ratios attributable to *Obp50a*, and sex-specific effects on starvation stress resistance attributable to *Obp50d*. Thus, there is functional diversification within the *Obp50* cluster with *Obp50a* contributing to development and *Obp50d* to stress resistance. The deletion–reinsertion approach we applied to the *Obp50* cluster provides a general paradigm for the genetic dissection of paralogs of multigene families.

## Introduction

Gene duplication followed by functional diversification represents a major mechanism for genome evolution, especially the evolution of large multigene families (reviewed by Long et al. 2013), such as chemoreceptors (Robertson et al. 2003; Croset et al. 2010; Hughes et al. 2018; Anholt 2020) and detoxification enzymes (Sezutsu et al. 2013; Good et al. 2014). In addition to subfunctionalization and neofunctionalization (Hahn 2009), multigene families may harbor functional redundancy, and this may account, in part, for the observation that functionally redundant multigene families may provide robustness to the transcriptome in the face of changing environmental conditions (Zhou et al. 2012). 

Insect odorant-binding proteins (OBPs) provide an example of a rapidly evolving multigene family (Sánchez-Gracia et al. 2009; Vieira and Rozas 2011). OBPs are small, secreted proteins with diverse amino acid sequences mostly characterized by six conserved cysteines (Hekmat-Scafe et al. 2002; Pelosi et al. 2014). Members of this family have been implicated in responses to pheromones (reviewed by Stengl 2010) and host plant odorants (reviewed by Anholt 2020). In *Drosophila melanogaster*, most of the 52 *Obp* genes occur in clusters distributed across the three major chromosomes, likely due to repeated tandem gene duplication. Behavioral (Swarup et al. 2011) and electrophysiological (Scheuermann and Smith 2019) studies have implicated several of these OBPs in modulating responses to odorants, although simultaneous CRISPR excision of four OBPs that are prominently expressed in the antenna did not affect electrophysiological responses upon exposure to odorants (Xiao et al. 2019). Whereas OBPs were thought to be primarily associated with olfactory responses (Pelosi et al. 2014; Larter et al. 2016), expression of OBPs in nonchemosensory tissues (McGraw et al. 2004; Findlay et al. 2008) suggests that some members of this family have evolved to acquire different functions. Association studies in wild derived lines of the *Drosophila melanogaster* Genetic Reference Panel (Mackay et al. 2012; Huang et al. 2014) identified two polymorphisms in *Obp19d* that were associated with lifespan (Arya et al. 2010). In addition, *Obp8a* and *Obp19c* are highly expressed in the male accessory gland and *Obp19c* is also expressed in ovaries (Findlay et al. 2008). Gene ontology enrichment analyses of coregulated transcripts revealed that transcripts associated with variation in *Obp19c* (designated as its “transcriptional niche”) implicate oviposition and postmating behavior (Arya et al. 2010).

Here, we report the genetic dissection of the *Obp50a–d* cluster of *D. melanogaster.* We selected this cluster because its organization is compact without intervening genes, its CRISPR-mediated excision results in viable offspring, and the functions of the four paralogs contained within this cluster are unknown. Members of this cluster show sexually dimorphic expression (Zhou et al. 2009) and in males are expressed at highest levels in testes (Roy et al. 2010; Hu et al. 2017; Leader et al. 2018; Thurmond et al. 2019), suggesting that paralogs of the *Obp50* family may have acquired functions that are unrelated to chemoreception.

We excised the *Obp50a–d* cluster using CRISPR/*Cas9* technology while introducing a *PhiC31* viral integration site at the endogenous locus. This enabled us to reinsert versions of the cluster in which all four, none, or one of the paralogs were intact, whereas the rest were rendered inactive through the introduction of premature termination codons. This in turn enabled us to isolate the functions of each paralog while accounting for their functional redundancy, quantify the extent to which they had diverged in function, and measure the magnitude and direction of epistatic interactions among them.

## Results

### Generation of an *Obp50a–d* Knockout Line and Reinsertion of Individual Functional Paralogs at Their Endogenous Location

**Fig. 1.**
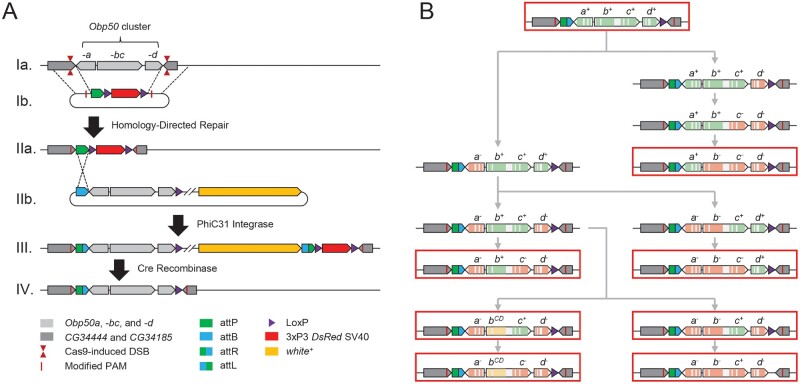
Schematic of genome editing strategy used to generate reinsertion lines. (*A*) Replacement of the *Obp50a–d* cluster. Cas9 was directed to induce double-stranded breaks (DSBs) on either side of the *Obp50a–d* cluster in the wild-type genome (*Ia*) whereas a *pDsRed*-*attP* repair template containing homology to either side (*Ib*) was coinjected, enabling homology-directed repair to replace the cluster with the *attP*-*LoxP*-*DsRed*-*LoxP* cassette. A single nucleotide substitution in each protospacer adjacent motif (PAM) was necessary to prevent Cas9 from cleaving the template, which resulted in *CG34444* G233A and *CG34185* G113A substitutions. Positive transformants (*IIa*) were crossed to a PhiC31 integrase-expressing line and injected with *pattB*-*Obp50ad*-*LoxP*-*white*^+^ vectors containing modified versions of the *Obp50a–d* cluster (*IIb*), which integrated in their entirety into the *attP* locus. The resulting chromosome (*III*) was passed through a Cre-expressing line to eliminate the more than 7 kb of unwanted sequence between the three unidirectional LoxP sites, leaving only a 60-bp attR and 34-bp LoxP flanking the reinserted cluster at the 3′ ends of all adjacent genes (*IV*). (*B*) Construction of *pattB* vectors with the eight reinsertion genotypes. After cloning the wild-type (“+” allele) *Obp50a–d* cluster (top) into the multiple cloning site (MCS) of the *pattB*-*MCS*-*LoxP*-*white^+^* vector, a series of site-directed mutagenesis reactions (gray arrows) were performed to either inactivate paralogs with premature termination codons (PTCs; “−” allele) or induce missense mutations in four conserved cysteines and a conserved alanine in *Obp50b* (“CD” allele). Exons of *Obp50a–d* paralogs are colored to indicate whether the respective gene is functional (green), inactivated by PTCs (red), or has four conserved cysteines and an alanine substituted (yellow). The substituted cysteines correspond to C2, C3, C5, and C6 from Hekmat-Scafe et al. (2002). Red outlines indicate the final vectors which produced the eight reinsertion genotypes.

We generated transgenic lines in the Canton S (B) genetic background to functionally dissect the *Obp50a–d* gene cluster. First, we used CRISPR/*Cas9* in conjunction with a homology-directed repair template to replace the wild-type *Obp50a–d* cluster with an *attP-LoxP-DsRed-LoxP* cassette (Gratz et al. 2014) (fig. 1*A* and supplementary table S1 and data file 1, Supplementary Material online). We then used the integrated *attP* site to introduce eight *pattB-Obp50ad-LoxP* plasmids, each with a unique version of the cluster (fig. 1*B*), into the endogenous locus (fig. 1*A*). We obtained 26 “reinsertion” lines of these eight genotypes (supplementary table S2 and data file 2, Supplementary Material online). This resulted in genotypes in which all, none, or one of the paralogs were functionally intact.

To account for possible redundancy between paralogs, the genotypes were designed to determine which functions each was sufficient to perform when all others were inactivated. In most cases, genes were inactivated via two consecutive premature termination codons (PTCs; “−” allele) early in their coding sequence. However, because the start codon of *Obp50c* is internal to the *Obp50b/c* bicistronic transcript, introducing PTCs into *Obp50b* could jeopardize the expression of *Obp50c*, since nonsense-mediated decay could degrade the transcript (Hug et al. 2016) or impede translation initiation of *Obp50c*. Therefore, we generated another genotype with a functional *Obp50c* (*Obp50a^−^b^CD^c^+^d^−^*) in which *Obp50b* was inactivated by substitution mutations (“CD” allele) in four conserved cysteines (C68S, C72S, C148S, and C158S) and a conserved alanine (A152P). As the functional consequences of amino acid substitutions in these conserved residues are unknown, we generated an additional genotype (*Obp50a^−^b^CD^c^−^d^−^*) with these same mutations.

Although *PhiC31* transgenesis causes the *pattB* plasmids to be integrated in their entirety, including the 7-kb backbone, the rearrangement of the multiple cloning site and existing *loxP* sequence (Bischof et al. 2013) allows virtually all extraneous sequence to be removed while leaving the reinserted *Obp50a–d* cluster intact by crossing positive transformants to a *Cre*-expressing line (fig. 1*A* and supplementary figs. S1 and S2, Supplementary Material online). The *Obp50a–d* cluster is especially amenable to this strategy, since orientation of the *Obp50a* and *Obp50d* genes is such that the remaining 61 and 39 bp of extraneous sequence up- and downstream of the cluster, respectively, are past the 3′ ends of all adjacent genes and are, therefore, less likely to interfere with any promoter or regulatory sequence (Roy and Singer 2015) (fig. 1).

### Phenotypic Effects of *Obp50a–d* Paralogs Show Functional Diversification in Females and Redundancy in Males

**Fig. 2.**
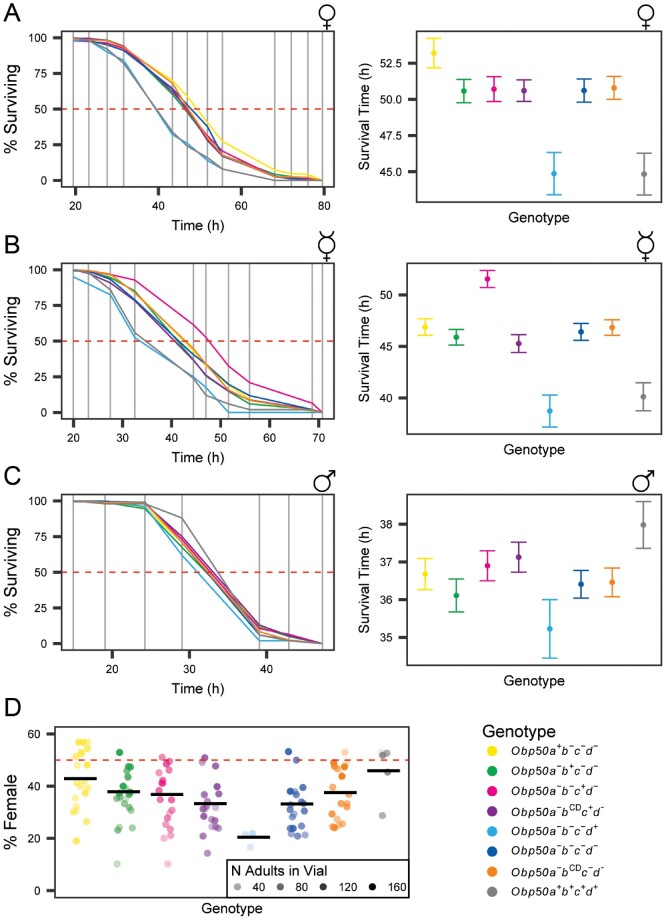
*Obp50a–d* genes show sex-specific functional diversification in starvation resistance and sex ratio. (*A-C*) Plots of % surviving over time (left) and average time of death versus genotype (right) of mated (*A*) and virgin (*B*) females and mated males (*C*) under starvation conditions. The horizontal dashed red line indicates 50% surviving. Vertical gray lines indicate observation times. Error bars are SEM. (*D*) Plot of % female offspring. Dots indicate independent vials in which flies of the respective genotype were allowed to lay eggs, where the opacity corresponds to the number of adult progeny which emerged from the respective vial. Black horizontal bars indicate the mean % female of the respective genotype’s vials weighted by the number of adult offspring per vial. Horizontal dashed red line indicates 50% female.

In contrast to many members of the *Obp* gene family, which function in chemosensation, *Obp50* paralogs are expressed in metabolic tissues and male reproductive organs (supplementary data file 3, Supplementary Material online). We examined a range of metabolic and reproductive phenotypes to assess functional diversification of the *Obp50a–d* cluster. We did not observe significant differences between the *Obp50a^+^b^+^c^+^d^+^* and *Obp50a^−^b^−^c^−^d^−^* genotypes for chill coma recovery time, startle response, or copulation latency or duration (supplementary table S3, Supplementary Material online). We did observe a significant difference between these genotypes for starvation resistance of mated females, such that the flies which possessed a fully intact *Obp50a–d* cluster (genotype *Obp50a^+^b^+^c^+^d^+^*) were significantly more sensitive to starvation stress than the complete knockout *Obp50a^−^b^−^c^−^d^−^* (*P *<* *0.0001, *n *≥* *89 flies/line, logistic regression) (supplementary data files 4 and 5, Supplementary Material online). Repeating the experiment with all eight genotypes (supplementary data files 4 and 5, Supplementary Material online) confirmed the difference between the *Obp50a^+^b^+^c^+^d^+^* and *Obp50a^−^b^−^c^−^d^−^* genotypes (*P *=* *0.0004, *n *≥* *49) and showed an almost identical difference between *Obp50a^−^b^−^c^−^d^+^* and *Obp50a^−^b^−^c^−^d^−^* (*P *=* *0.0005, *n *≥* *49; fig. 2*A*). *Obp50a^+^b^+^c^+^d^+^* and *Obp50a^−^b^−^c^−^d^+^* had nearly equivalent mean survival times (44.6 and 44.7 h, respectively) and were not significantly different from each other (*P*_Holm_* *=* *1, *n *≥* *50), which indicates that the difference in resistance to starvation stress between the intact and deleted *Obp50a–d* cluster could be attributed to a functional *Obp50d* allele, as no other comparisons were significant.

Virgin females yielded similar results, except that the *Obp50a^−^b^−^c^+^d^−^* genotype was more resistant to starvation compared with *Obp50a^−^b^−^c^−^d^−^* (*P *<* *0.0001, *n *≥* *33; fig. 2*B*). *Obp50a^−^b^−^c^+^d^−^* also survived longer than *Obp50a^−^b^CD^c^+^d^−^* virgin female flies (*p*_Holm_* *<* *0.0001, *n *≥* *28). As no difference was seen between the *Obp50a^−^b^CD^c^−^d^−^* and *Obp50a^−^b^−^c^−^d^−^* genotypes (*P*_Holm_* *=* *1, *n *≥* *29), these results suggest an epistatic interaction between the functional *Obp50c* gene and the *Obp50b^CD^* allele, and that this interaction depends on both sex and mated status.

The *Obp50a^+^b^+^c^+^d^+^* genotype also differed significantly from *Obp50a^−^b^−^c^−^d^−^* in the mated male starvation stress resistance assay (*P *=* *0.0457, *n *≥* *49), but in males *Obp50a^+^b^+^c^+^d^+^* was the most resistant genotype, in contrast to the results seen in females (fig. 2*C*). Moreover, *Obp50a^−^b^−^c^−^d^+^* was significantly different from *Obp50a^+^b^+^c^+^d^+^* (*P*_Holm_* *=* *0.0411, *n *≥* *50) and had the lowest mean survival time of all the genotypes, but was not significantly different from *Obp50a^−^b^−^c^−^d^−^* (*P *=* *0.1652, *n *≥* *49).

To test whether the observed differences in starvation resistance could be accounted for by a greater investment in reproduction-related processes (Harbison et al. 2004; Wayne et al. 2006), we maintained vials in which mated females were allowed to lay eggs overnight before being submitted to starvation and counted their adult progeny. There was no correlation between starvation resistance and fecundity (*P *=* *0.2412, sum of squares* *=* *325.8, *F* ratio* *=* *1.38, Ordinary Least Squares test, *n *≥* *7 vials/line). We also assessed whether increased starvation resistance might be due to differences in larval fat content (Chippindale et al. 1996; Djawdan et al. 1998; Aguila et al. 2007) using a buoyancy assay on wandering stage larvae (Hazegh and Reis 2016), but found no significant correlation between genotype and inferred larval fat content (*P *=* *0.4801, *n *=* *6 vials/line; supplementary table S3, Supplementary Material online).

In addition to antagonistic effects between males and females on resistance to starvation stress, we observed differences in sex ratio among the genotypes as measured by the percent of female offspring (fig. 2*D*) (supplementary data files 4 and 6, Supplementary Material online). The *Obp50a^+^b^+^c^+^d^+^* genotype had the highest percent of females (46%) and differed significantly from *Obp50a^−^b^−^c^−^d^−^* (32%, *P *<* *0.0001, *n *≥* *4 vials/line). The female sex ratio of *Obp50a^+^b^−^c^−^d^−^* (42%) was also significantly different from *Obp50a^−^b^−^c^−^d^−^* (*P *<* *0.0001, *n *≥* *4) but not from *Obp50a^+^b^+^c^+^d^+^* (*P*_Holm_* *=* *0.4631, *n *≥* *4), indicating that the reinsertion of intact *Obp50a* was sufficient to restore a nearly normal sex ratio. *Obp50d* had the opposite effect, however, as *Obp50a^−^b^−^c^−^d^+^* produced even fewer females than *Obp50a^−^b^−^c^−^d^−^* (20%, *P *=* *0.0085, *n *≥* *3). Given that the number of adult progeny in each vial correlated significantly with the percent of females (*P *<* *0.0001) and most (108/125, 86%) vials had fewer females than males, we concluded that differences in sex ratio were attributed to differences in female survival during development.

Although the reinsertion of a functional *Obp50c* did not increase the proportion of females when *Obp50b* was inactivated with PTCs (34%, *P *=* *0.1764, *n *≥* *4), it decreased the female/male sex ratio in the presence of the *Obp50b^CD^* allele (*P*_Holm_* *=* *0.0206, *n *≥* *4). Since *Obp50a^−^b^CD^c^−^d^−^* had a higher percentage of females than *Obp50a^−^b^−^c^−^d^−^* (38%, *P*_Holm_* *=* *0.0041, *n *≥* *4), *Obp50b^CD^* partially restored the sex ratio in the absence of *Obp50c*. This implies an antagonistic relationship between *Obp50c* and *Obp50b^CD^* and indicates that *Obp50b^CD^* may have residual or altered function.

For each of the above phenotypes, the extent of functional diversification between the four paralogs was determined by testing for differences between the *Obp50a^+^b^−^c^−^d^−^*, *Obp50a^−^b^+^c^−^d^−^*, *Obp50a^−^b^−^c^+^d^−^*, and *Obp50a^−^b^−^c^−^d^+^* genotypes, with the null hypothesis that the four paralogs were functionally similar, or redundant. These tests showed the paralogs to have diverse effects on female starvation resistance (mated: *P *=* *0.0002, *n *≥* *25 flies/line; virgin: *P *<* *0.0001, *n *≥* *27) and sex ratio (*P *<* *0.0001, *n *≥* *3 vials/line), while having redundant effects (*P *=* *0.1392, *n *≥* *49 flies/line) on male starvation resistance.

If the paralogs have additive phenotypic effects, we expect that the effect of the full *Obp50a–d* cluster (estimated by the difference between the *Obp50a^+^b^+^c^+^d^+^* and *Obp50a^−^b^−^c^−^d^−^* genotypes) will equal to the sum of the effects of the individual paralogs (which were each estimated by the difference between *Obp50a^−^b^−^c^−^d^−^* and the respective single paralog reinsertion genotype, whether *Obp50a^+^b^−^c^−^d^−^*, *Obp50a^−^b^+^c^−^d^−^*, *Obp50a^−^b^−^c^+^d^−^*, or *Obp50a^−^b^−^c^−^d^+^*). Epistasis occurs if this is not the case. We inferred that the effects of the paralogs were additive in each of these assays, with *P* values of 0.6965 (*n *≥* *25 flies/line), 0.2087 (*n *≥* *27), 0.1194 (*n *≥* *49), and 0.087 (*n *≥* *3 vials/line) for mated female, virgin female, and male starvation resistance, and sex ratio, respectively.

### Transcriptional Profiling of the *Obp50a–d* Reinsertion Lines Shows Coregulated Transcripts Expressed in the Pupal Fat Body and Male Reproductive Tissues

**Fig. 3.**
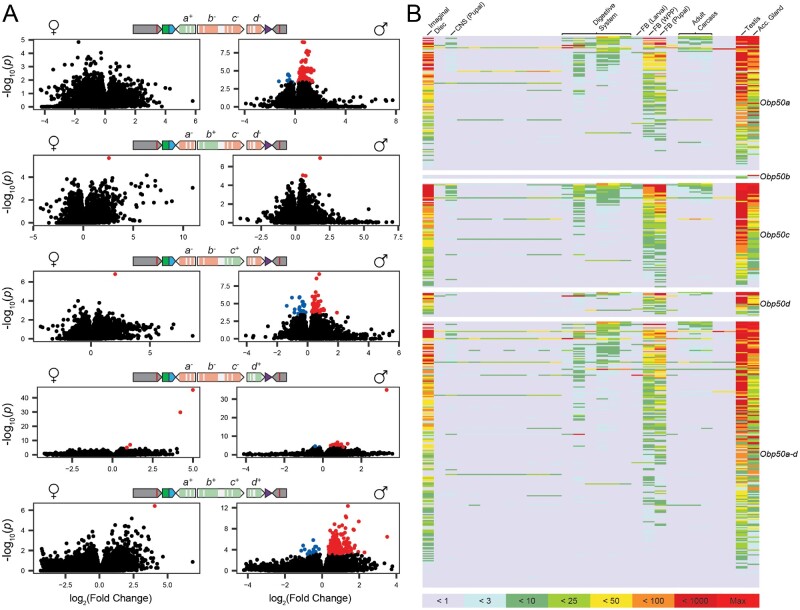
*Obp50a–d* paralogs selectively up-regulate male transcripts expressed in reproductive and metabolic tissues. (*A*) Volcano plots of female (left) and male (right) contrasts between select genotypes (indicated by the schematic chromosomes) and the *Obp50a^−^b^−^c^−^d^−^* genotype in which all paralogs have been inactivated. Black dots represent genes which did not pass the FDR threshold of 0.05; blue and red dots represent genes which were significantly down- and up-regulated in the presence of the functional paralog(s), respectively, collectively comprising the corresponding paralog(s)’ “transcriptional niche.” (*B*) Tissue expression heatmaps constructed from modENCODE RNAseq data of male differentially expressed genes from panel (*A*). The corresponding transcriptional niche for each heatmap is indicated on its right. Colors correspond to the read count bins in the key below.

We next performed transcriptome profiling to assess to what extent reinsertion of the *Obp50a–d* paralogs affects their “transcriptional niches,” defined as the coregulated ensembles of differentially expressed genes (DEGs) upon altered expression of a focal paralog or set of paralogs (Arya et al. 2010) (supplementary data files 4 and 7–11, Supplementary Material online). We performed RNAseq on whole flies and examined contrasts between the full cluster knockout (*Obp50a^−^b^−^c^−^d^−^*) and each of the four single paralog reinsertion genotypes (*Obp50a^+^b^−^c^−^d^−^*, *Obp50a^−^b^+^c^−^d^−^*, *Obp50a^−^b^−^c^+^d^−^*, *Obp50a^−^b^−^c^−^d^+^*) as well as the genotype with a fully intact *Obp50a–d* cluster (*Obp50a^+^b^+^c^+^d^+^*) to estimate the corresponding transcriptional niches within each sex. (*Obp50a^−^b^CD^c^+^d^−^* and *Obp50a^−^b^CD^c^−^d^−^* were not compared to estimate the transcriptional niche of *Obp50c* for reasons discussed below.) An FDR threshold of 0.05 was used to determine significance. We observed pronounced sexual dimorphism, with most effects on gene expression in males (fig. 3*A* and supplementary fig. S4, Supplementary Material online). There were only seven DEGs in all five female transcriptional niches combined, all of which were up-regulated in the presence of their respective paralogs. The male transcriptional niches were larger, with *Obp50a–d* having the most coregulated transcripts (226, 92% upregulated), followed by *Obp50a* (114, 93% upregulated), *Obp50c* (88, 75% upregulated), *Obp50d* (21, 90% upregulated), and *Obp50b* (3, 100% upregulated).

Based on MODEncode data, genes belonging to transcriptional niches of *Obp50* paralogs in males were enriched for expression in the testes, accessory glands, imaginal disc, and pupal and prepupal fat body, with some notable expression in the digestive system (fig. 3*B* and supplementary data file 12, Supplementary Material online). Since this pattern mirrors the reported expression of the *Obp50a–d* paralogs themselves (supplementary data file 3, Supplementary Material online), these observations confirm that promoters and regulatory regions were not disrupted.

We next assessed the effect of the *Obp50b^CD^* allele (supplementary fig. S4*B*, Supplementary Material online). Although both *Obp50a^−^b^+^c^−^d^−^* and *Obp50a^−^b^CD^c^−^d^−^* had minimal effects compared with *Obp50a^−^b^−^c^−^d^−^* (supplementary fig. S4*B* top, middle, Supplementary Material online), a comparison between them revealed 47 DEGs in females and 141 in males (15% and 59% of which were upregulated in *Obp50a^−^b^+^c^−^d^−^*, respectively; supplementary fig. S4*B* bottom, Supplementary Material online). This indicated that the point mutations introduced in *Obp50b^CD^* result in changes in the gene’s transcriptional niche. We therefore deem the comparison between the *Obp50a^−^b^−^c^+^d^−^* and *Obp50a^−^b^−^c^−^d^−^* genotypes to be a better estimate of the function of *Obp50c* than the comparison between *Obp50a^−^b^CD^c^+^d^−^* and *Obp50a^−^b^CD^c^−^d^−^*.

We also examined the differences between the transcriptional niches of *Obp50b^CD^* and *Obp50c* (supplementary fig. S4*C*, Supplementary Material online). *Obp50b^CD^* reduced by half the number of DEGs attributable to *Obp50c* in males (from 88 to 44) and shifted the percent of up-regulated genes in the presence of *Obp50c* from 75% to 14% (supplementary fig. S4*C* top right, middle right, Supplementary Material online). This implies that the magnitude and direction of the effects of *Obp50c* depend on *Obp50b^CD^*. Moreover, the effect of *Obp50b^CD^* in males depends on an intact *Obp50c*, as many more DEGs are attributable to its function when an intact *Obp50c* is present (234, 12% of which were upregulated; supplementary fig. S4*C* bottom right, Supplementary Material online) compared with a dysfunctional allele (1, which was upregulated; supplementary fig. S4*B* middle right, Supplementary Material online). This evidence of an active effect of the *Obp50b^CD^* allele on the transcriptome further justifies our decision not to use *Obp50b^CD^* as a proxy for an inactive *Obp50b*. In females, however, the effects of *Obp50b^CD^* and *Obp50c* could not be compared due to the almost complete lack of DEGs in any of these contrasts (supplementary fig. S4*B* middle left and S4*C* left, Supplementary Material online).

### Analysis of Transcriptional Niches Reveals Functional Redundancy, Diversification, and Epistasis among Paralogs

**Fig. 4.**
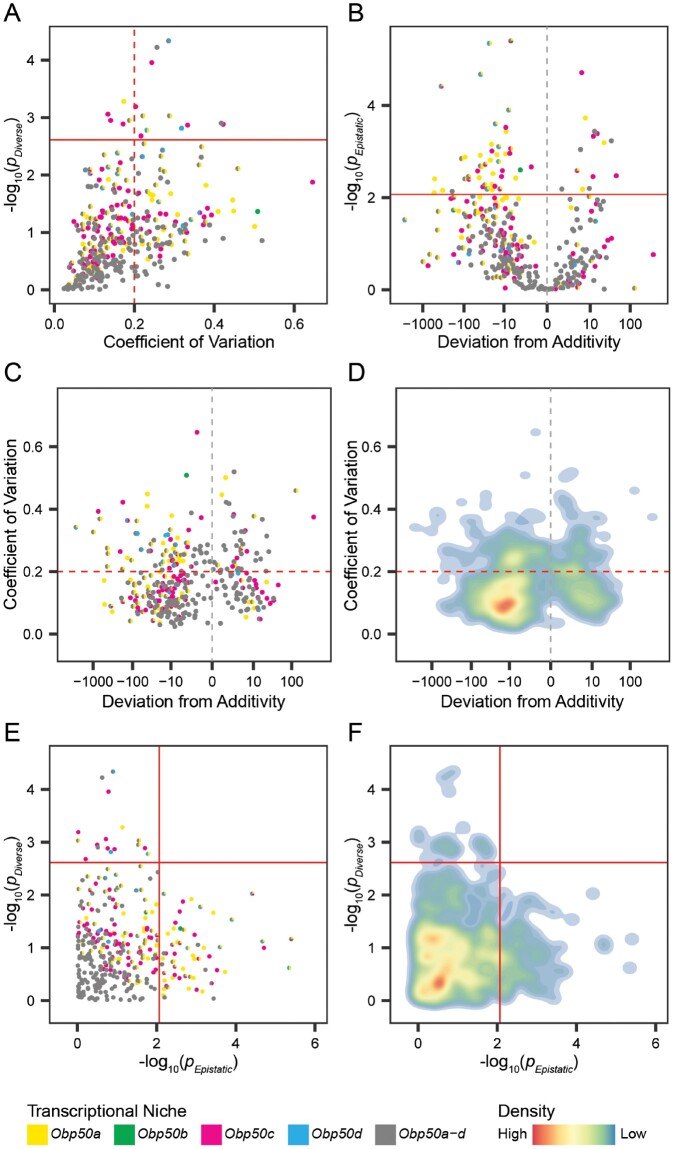
Male transcriptional niches of *Obp50a–d* paralogs demonstrate considerable redundancy and additivity. (*A*) Plot of each gene’s −log_10_(*P*) in the test for functional diversification (ANOVA model $Y=\mathrm{Genotype}+\mathrm{Line}\left(\mathrm{Genotype}\right)+\;\varepsilon$ of the *Obp50a^+^b^−^c^−^d^−^*, *Obp50a^−^b^+^c^−^d^−^*, *Obp50a^−^b^−^c^+^d^−^*, and *Obp50a^−^b^−^c^−^d^+^* genotypes, in which Y is observed expression), versus the coefficient of variation between the least squares means of these genotypes’ expression. Larger values of −log_10_(*P*) indicate increasingly significant diversification. (*B*) Plot of each gene’s −log_10_(*P*) versus estimate in the test for epistasis (see Materials and Methods). Larger values of −log_10_(*P*) indicate increasingly significant epistasis. (*C*) Plot of coefficient of variation from (*A*) versus the epistasis estimate from (*B*). (*D*) Density plot of (*C*). (*E*) Plot of −log_10_(*P*) from (*A*) versus −log_10_(*P*) from (*B*). (*F*) Density plot of (*E*). The solid red line indicates FDR* *=* *0.05. The dashed red line indicates a coefficient of variation of 0.2, and the dashed gray line indicates a deviation from additivity of 0. For clarity, the extreme outlier *CG13177*, belonging to the *Obp50d* transcriptional niche, is not shown (for all genes and both sexes see supplementary fig. S5, Supplementary Material online).

We quantified the extent of redundancy versus diversification among the *Obp50a–d* paralogs by testing for a difference in expression between the *Obp50a^+^b^−^c^−^d^−^*, *Obp50a^−^b^+^c^−^d^−^*, *Obp50a^−^b^−^c^+^d^−^*, and *Obp50a^−^b^−^c^−^d^+^* genotypes for each of the genes which belonged to at least one of the five transcriptional niches. Overlapping effects of paralogs on their respective transcriptional niches reflects redundancy, whereas distinct transcriptional niches is considered indicative of diversification. We assessed differences in the expression of transcripts between the *Obp50a^+^b^−^c^−^d^−^*, *Obp50a^−^b^+^c^−^d^−^*, *Obp50a^−^b^−^c^+^d^−^*, and *Obp50a^−^b^−^c^−^d^+^* genotypes (fig. 4 and supplementary fig. S5 and data file 14, Supplementary Material online), separately by sex. We used an FDR cutoff of 0.05 to define genes on which the *Obp50a–d* paralogs had redundant (FDR ≥ 0.05) or functionally diverse (FDR* *< 0.05) effects.

To address the possibility that the effects of the paralogs could appear redundant merely due to low statistical power, we sought to quantify the variance between the effects of the four paralogs on the expression of each gene, with lower variance between their effects corresponding to greater functional redundancy. For each of these transcriptional niche genes we calculated the coefficient of variation (CoV) of the least squares means of the four genotypes used in the test for functional diversification, with CoV being defined as the ratio of the standard deviation to the mean.

The paralogs had redundant effects on the vast majority (326, 95%) of the 344 male transcriptional niche genes, and 224 (65%) had a CoV* *<* *0.2 (fig. 4*A* and supplementary fig. S5*A* right, Supplementary Material online); in females, these values were 1/7 (14%) and 0/7, respectively (supplementary fig. S5*A* left, Supplementary Material online). *CG13177* is the extreme outlier in the *Obp50d* transcriptional niche of both females and males and is the only gene common to a transcriptional niche in both sexes. In males, its CoV was 1.47, more than twice that of the next nearest gene, and in females it had the highest CoV, as well as the most significant *P* value. *CG13177* encodes a transcript of unknown function expressed in the digestive system. A BLASTn search (Zhang et al. 2000) reveals alignment of *CG13177* with predicted neuropeptide-like proteins 30 and 31 in related *Drosophila* species (supplementary data file 15, Supplementary Material online).

Transcriptional niche genes for which the paralogs had epistatic effects were defined as noted above: the effect of the full *Obp50a–d* cluster deviated significantly (FDR* *<* *0.05) from the sum of the effects of the individual paralogs (supplementary data file 14, Supplementary Material online). This was estimated separately for each transcriptional niche gene by comparing the difference between the expression of the *Obp50a^+^b^+^c^+^d^+^* and *Obp50a^−^b^−^c^−^d^−^* genotypes to the sum of the differences between the expression of *Obp50a^−^b^−^c^−^d^−^* and each of the *Obp50a^+^b^−^c^−^d^−^*, *Obp50a^−^b^+^c^−^d^−^*, *Obp50a^−^b^−^c^+^d^−^*, and *Obp50a^−^b^−^c^−^d^+^* genotypes. We divided epistasis into “enhancing” and “suppressing” categories, enhancing when the effect of the complete intact cluster was greater than the sum of the effects of the individual paralogs, and suppressing when the effects of individual paralogs exceeded that of the full cluster. The *Obp50a–d* paralogs had epistatic effects on 61 (18%) of the 344 male transcriptional niche genes, 14 (23%) of which were enhancing and 47 (77%) were suppressing (fig. 4*B* and supplementary fig. S5*B* right, Supplementary Material online). Epistatic effects were detected in 5/7 (71%) of female transcriptional niche genes, all but one of which (80%) were suppressing (supplementary fig. S5*B* left, Supplementary Material online). *CG13177* was an extreme outlier in this regard as well in both females and males.

To examine the relationship between the redundancy/diversification and additivity/epistasis axes, we plotted the estimates of their effect sizes (i.e., CoV and deviation from additivity, respectively; fig. 4*C* and *D*; supplementary fig. S5*C*, Supplementary Material online) against their statistical significance (fig. 4*E* and *F*; supplementary fig. S5*D* and data file 14, Supplementary Material online). In males, the approximately symmetrical distribution of CoV (fig. 4*C* and *D*) indicated that there was no clear dependence of CoV on the direction of epistasis. Most of the effects of the paralogs in males were both redundant and additive, accounting for 266 (77%) of the 344 transcriptional niche genes, with −log_10_(*P*) values concentrated in the areas of high redundancy and additivity, well below the statistical significance threshold (fig. 4*E* and *F*; supplementary fig. S5*D*, Supplementary Material online). Of note is the extent to which the areas of maximal redundancy and additivity are enriched for the *Obp50a–d* transcriptional niche genes (bottom left corner of fig. 4*E*), consistent with their disproportionate occupation of areas of low CoV and small deviations from additivity (fig. 4*C*). The remaining male effects were almost all either redundant and epistatic (60/344, 17%) or additive and divergent (17/344, 5%). The sole gene on which the paralogs exerted both divergent and epistatic effects was *CG13177* (supplementary fig. S5*C* and *D* right, Supplementary Material online). This pattern is reversed in females, with the paralogs not having redundant and additive effects on any genes, redundant and epistatic effects on a single gene (14%), divergent and additive effects on 2/7 (29%) genes, and divergent and epistatic effects on 4/7 (57%) genes (supplementary fig. S5*D* left, Supplementary Material online).

**Fig. 5.**
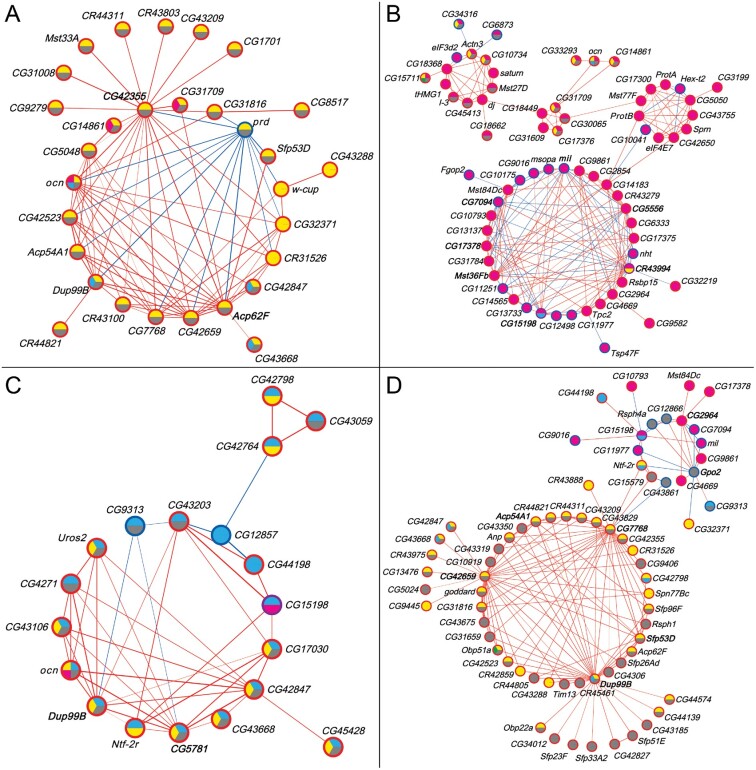
Coexpression networks of male *Obp50a* (*A*), *Obp50c* (*B*), *Obp50d* (*C*), and *Obp50a*, -*b*, -*c*, -*d*, and *Obp50a–d* (*D*) transcriptional niches (see fig. 3). Node fill color indicates membership in the corresponding transcriptional niche (see fig. 4). Node border color indicates whether the gene is up- (red) or down- (blue) regulated compared with *Obp50a^−^b^−^c^−^d^−^* in the niche(s) to which it belongs; purple indicates that the gene is up-regulated in one and down-regulated in another. Edge color indicates positive (red) or negative (blue) correlation between genes, with the strength of the correlation proportional to the edge width. Gene labels of nodes with the most edges (top 10%) are bolded. See also supplementary table S4, Supplementary Material online, which summarizes the number of genes common to all networks (redundancy), as well as the number of genes that are specific to each paralog network (diversification).

We constructed coexpression networks for the *Obp50a*, *Obp50c*, and *Obp50d* male transcriptional niche genes separately (fig. 5*A–*C; *Obp50b* had too few genes in its niche for construction of an independent network), as well as for all transcriptional niche genes together (fig. 5*D* and supplementary data files 16 and 17, Supplementary Material online). Consistent with previous results, most genes in the networks were up-regulated in the presence of at least one intact paralog (see fig. 3). Of the genes common to more than one transcriptional niche, virtually all agreed in their direction; accordingly, most correlations between genes were positive.

There is substantial overlap between the *Obp50a–d* niche and that of *Obp50a* (26/30, 87%) and *Obp50d* (13/19, 68%) in the respective networks (fig. 5*A* and *C*), consistent with the finding of extensive redundancy and additivity between these paralogs (see fig. 4). This is in contrast to *Obp50c*, whose overlap with *Obp50a–d* was less extensive (15/68, 22%) and unevenly distributed within its network (fig. 5*B*). This uneven distribution of overlap is suggestive of subnetworks with discrete levels of redundancy and diversification. The uniqueness of *Obp50c* can be clearly seen in the full network (fig. 5*D*), in which all *Obp50c* transcriptional niche genes are confined to a single region.

Concordant with the tissue expression and gene ontology results (see fig. 3*B*), the networks consisted of accessory proteins, seminal fluid proteins, male-specific transcripts, and other genes with known functions in male reproduction. Accordingly, notable hub genes, defined as being in the top 10% of network genes with the most significant correlations (*i.e*., edges), are all expressed in the male accessory gland or testes, including *Acp62F* (fig. 5*A*), *Mst36Fb* (fig. 5*B*), *Dup99B* (fig. 5*C* and *D*), and *Gpo2* (fig. 5*C*).

**Fig. 6.**
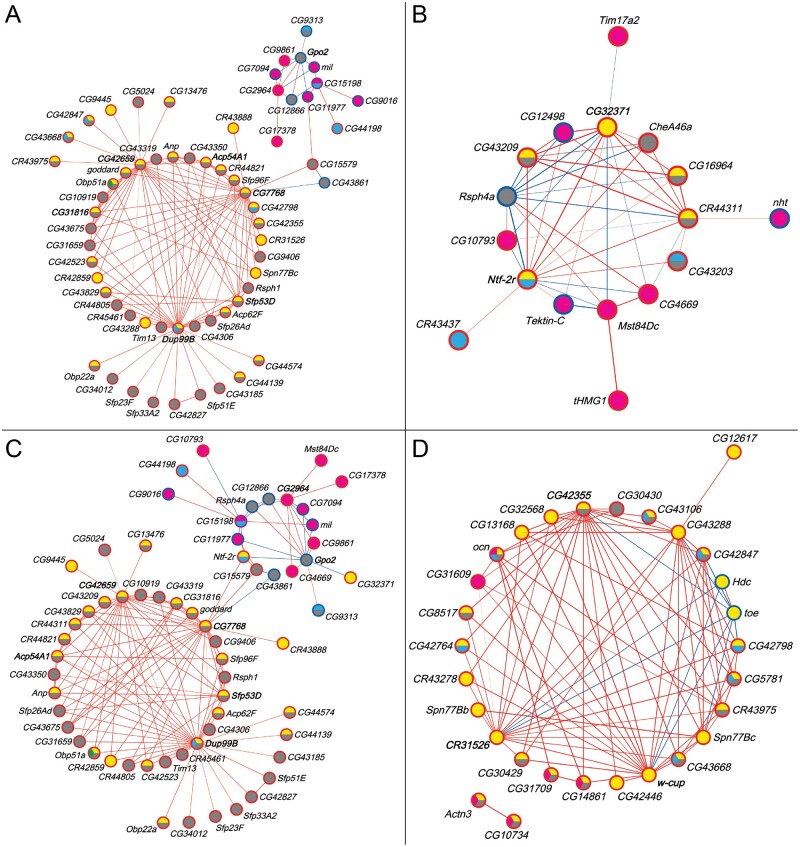
Coexpression networks of male transcriptional niche genes on which the *Obp50a–d* paralogs have redundant (*A*), diverse (*B*), additive (*C*), and epistatic (*D*) effects. Node fill color indicates membership in the corresponding transcriptional niche (see fig. 4). Node border color indicates whether the gene is up- (red) or down- (blue) regulated compared with *Obp50a^−^b^−^c^−^d^−^* in the niche(s) to which it belongs; purple indicates that the gene is up-regulated in one and down-regulated in another. Edge color indicates positive (red) or negative (blue) correlation between genes, with the strength of the correlation proportional to the edge width. Gene labels of nodes with the most edges (top 10%) are bolded. See also supplementary table S4, Supplementary Material online, which summarizes the number of genes common to all networks (redundancy), as well as the number of genes that are specific to each paralog network (diversification).

We also constructed coexpression networks of the male transcriptional niche genes on which the *Obp50a–d* paralogs had redundant, diverse, additive, and epistatic effects (fig. 6*A–*D, respectively; see supplementary data files 16 and 17, Supplementary Material online). The redundant and additive networks were similar in structure and content to each other and to the network of all transcriptional niche genes (fig. 5*D* and supplementary table S4, Supplementary Material online). This is consistent with the previous finding that the paralogs exerted redundant and additive effects on most genes (see fig. 4). Genes within the *Obp50a–d* niche were notably more prevalent in the redundant (46/62, 74%) and additive (45/62, 73%) networks compared with the diversified (6/17, 35%) and epistatic (14/29, 48%) networks. Hub genes included *Dup99B*, *Acp54A1*, *Sfp53D*, and *Gpo2* (fig. 6*A* and *C*), *Ntf-2r* (fig. 6*B*), and *w-cup* (fig. 6*D*), again all expressed in male reproductive organs.

## Discussion

Gene duplications relieve evolutionary constraints on daughter genes, thereby allowing rapid expansion of large multigene families, such as the cytochrome P450 family dedicated to detoxification of xenobiotics (Sezutsu et al. 2013; Good et al. 2014) and families of chemoreceptors for the localization of food, predators, and mating partners (Robertson et al. 2003; Croset et al. 2010; Hughes et al. 2018; Anholt 2020). Insect OBPs are a diverse family of proteins, initially discovered as pheromone binding proteins (Vogt and Riddiford 1981) and annotated based on conservation of cysteine residues (Hekmat-Scafe et al. 2002). Whereas OBPs are implicated in olfaction, presumably by transferring hydrophobic odorants to their membrane-bound receptors, several members of the *Obp* family have functions other than chemoreception (Maleszka et al. 2007; Findlay et al. 2008; Takemori and Yamamoto 2009; Costa-Da-Silva et al. 2013; Heavner et al. 2013; Ishida et al. 2013; Marinotti et al. 2014; Wang et al. 2015; Zhu et al. 2016; Sun et al. 2018; reviewed in Pelosi et al. 2018). Gene products of the *Obp50* cluster are expressed in metabolic tissues and prominently in male testis and accessory gland (Roy et al. 2010; Hu et al. 2017; Leader et al. 2018; Thurmond et al. 2019), where they likely function as carriers for yet unidentified lipophilic compounds. All four *Obp50* paralogs have syntenic orthologs among species of the *melanogaster* group (*D. melanogaster, D. sechellia, D. yakuba, D. erecta*, and *D. ananassae*) and *D. willistoni* (Sánchez-Gracia et al. 2009; flybase.org), whereas only *Obp50a* and *Obp50b* have syntenic orthologs among species of the *obscura* group (*D. pseudoobscura* and *D. persimilis*). *Obp50a* has also syntenic orthologs in *D. mojavensis, D. virilis*, and *D. grimshawi. Obp50c* has a syntenic ortholog in *D. mojavensis* (flybase.org*).* Thus, it is likely that *Obp50a* is ancestral to the other orthologs and that *Obp50c* and *d* were lost in the *obscura* group. None of the *Obp50* paralogs have annotated syntenic orthologs in *D. suzukii*.

### A Deletion–Reinsertion Approach for the Genetic Dissection of Paralogs in Multigene Clusters

We designed a deletion–reinsertion strategy to genetically dissect the *Obp50* gene cluster by constructing transgenic lines in which all, none, or one of the *Obp50a–d* paralogs were functional. This design allowed analyses of organismal phenotypes and transcriptional niches associated with each paralog and quantification of redundancy, diversification, additivity, and epistasis among paralogs. Genetic dissection of paralogs of the *Obp50a–d* cluster with this deletion–reinsertion method highlights their complex evolutionary interrelationships, characterized by redundancy, diversification, and moderate epistasis. Diversification is evident from our observation that skewing of sex ratios could be attributed to *Obp50a*, whereas sex-specific effects on starvation stress resistance could largely be attributed to *Obp50d*, as well as *Obp50c* in virgin females. Effects on starvation resistance in females are likely due to transcriptional changes induced by inactivation of *Obp50* paralogs in the fat body and digestive system (fig. 3).This deletion–reinsertion approach provides a general paradigm for the genetic dissection of paralogs of multigene families in which functional diversification and epistasis can be quantified relative to any given phenotype.

### Sex-Specific Effects of *Obp50a–d* Paralogs

Analyses of individual *Obp50a–d* paralogs showed the largest effect on the male transcriptome, where genes involved in metabolism and reproduction are up-regulated in accordance with their highly enriched expression in the testes, accessory glands, and pupal fat body. Although members of the *Obp* gene family are present in seminal fluid (Findlay et al. 2008, 2009; Takemori and Yamamoto 2009; Sepil et al. 2019), the transfer of OBP50a–d to females during mating has not yet been reported. We postulate that these OBPs likely serve as carriers of lipophilic compounds which contribute to metabolic processes and regulate male reproduction. Many functions for which transcriptional niche genes in mated females were enriched, such as eggshell formation and embryogenesis, may be due at least partially to the upregulation of male seminal fluid proteins (Avila et al. 2011), but may also stem from a more direct role in these processes as seen with OBPs and related proteins in mosquitos (Costa-Da-Silva et al. 2013; Marinotti et al. 2014) and honeybees (Maleszka et al. 2007). The molecular mechanisms that regulate the dynamics of coregulated gene expression, *e.g.* upregulation or downregulation of gene expression when one paralog or more are inactivated, remain to be identified.

Our observations that the most significant organismal phenotypes were seen in females whereas the most pronounced effects on the transcriptome were seen in males is likely due to the different conditions under which these data were generated. The organismal-level effects on females are seen during development and under starvation stress, whereas for RNA sequencing adult flies grown under standard conditions were collected. Moreover, the effect of *Obp50c* on female starvation resistance was seen in virgin flies, whereas we only sequenced the transcriptome of mated flies.

### Maintenance of Redundancy by Dosage Effects and Transcriptional Buffering

The similarity between the effects of the individual *Obp50a–d* paralogs on the male transcriptome (fig. 4) suggests that the maintenance of these paralogs may at least partially be due to dosage effects (Kondrashov et al. 2002; Qian et al. 2010). This redundancy in males contrasts with the divergent effects of these paralogs on female organismal phenotypes. Given that redundant effects of duplicated genes likely reflect ancestral function, these results are compatible with the “out of the testis” hypothesis which posits that many new genes are initially expressed in the testis and later diversify into other tissues and functions (Kaessmann 2010).

We detected significant epistasis between the paralogs for almost one fifth (18%) of the male transcriptional niche genes. In most (77%) of these cases, all four paralogs together had less of an effect than would be expected from the effect of each paralog in isolation, indicating suppressing epistasis, which may contribute to maintenance of mutational robustness over long periods of evolutionary time (Gu et al. 2003; Conant and Wagner 2004; Yamamoto et al. 2009; Vandersluis et al. 2010). Purifying selection may not be able to purge loss-of-function mutations that accumulate in functionally compensatory paralogs (Ihmels et al. 2007), but the paralogs could attenuate transcriptional noise and thereby stabilize the phenotypic response (Nowak et al. 1997; Zhou et al. 2012; Saito et al. 2014). It is also possible that maintenance of redundancy is due to functional constraints (Vavouri et al. 2008) or that insufficient time has passed since duplication for complete neofunctionalization (Nowak et al. 1997).

### Redundant, Additive Effects of Duplicated Genes Are More Readily Apparent by Multiple-Paralog Knockouts

In line with previous studies, plotting measures of functional diversification and epistasis together did not reveal any clear correlation between them (Musso et al. 2008). However, we observed a salient pattern in which the most redundant and additive effects of the paralogs were only detectable in the intact *Obp50a–d* transcriptional niche. It is important to note that our measure of epistasis for a given transcript is calculated using the effect of the full *Obp50a–d* cluster, which is estimated by the difference between the expression of that transcript in the *Obp50a^+^b^+^c^+^d^+^* and *Obp50a^−^b^−^c^−^d^−^* genotypes. However, there is no *a priori* reason to suspect that this effect, if significant, will be similar to the sum of the effects of the individual paralogs, which is estimated by the sum of the differences between the expression of *Obp50a^−^b^−^c^−^d^−^* and each of the *Obp50a^+^b^−^c^−^d^−^*, *Obp50a^−^b^+^c^−^d^−^*, *Obp50a^−^b^−^c^+^d^−^*, and *Obp50a^−^b^−^c^−^d^+^* genotypes. This is because the sum of the effects of the individual paralogs could, in principle, assume any value (assuming no biological relationship) and cannot be predicted *a priori* by the effect of the full cluster. The pronounced additivity within the *Obp50a–d* niche, therefore, cannot be accounted for by a logical dependency inherent in our calculation of epistasis. We note, however, that our test for epistasis examines overall interactions among the *Obp50* paralogs. Further detailed analyses of epistasis are possible in the future by constructing the six genotypes with all possible pairs of paralogs and the four genotypes in which only one paralog is missing. These genotypes would enable inference of the role of sharing of regulatory elements in the paralog regions on epistatic interactions.

The detection of the most additive and redundant effects of the paralogs within the *Obp50a–d* niche emphasizes an underappreciated difficulty when studying additive effects of functionally similar genes, like duplicated genes: when a given additive effect is divided more evenly among multiple duplicates (Qian et al. 2010), the statistical power necessary to detect the effect of at least one of the duplicates is increased, assuming a constant level of statistical noise. In other words, similarity between the additive effects of the individual paralogs decreases the magnitude of the strongest single-paralog effect, which thereby decreases the likelihood that the effect of any one of the individual paralogs will be detected. Nonetheless, the more even distribution of additive effects among the paralogs does not change their sum, which is more likely to rise to the level of statistical significance than any of the single-paralog effects. This sum is represented by the difference between the *Obp50a^+^b^+^c^+^d^+^* and *Obp50a^−^b^−^c^−^d^−^* genotypes, thereby explaining why the most redundant effects that are additive are only detected in the *Obp50a–d* transcriptional niche. This further justifies our full-cluster deletion–reinsertion approach in which the collective effect of multiple paralogs can be measured.

## Materials and Methods

### Fly Husbandry

Unless otherwise indicated, Canton S (B) flies were reared on yeast-cornmeal-molasses-agar medium at 25°C, 60–75% relative humidity, and a 12 h light–dark cycle. Adult flies were 3–5 days old at the start of each assay and screened for morphological aberrations.

### Generation of CRISPR-Cas9 Excision and PhiC31 Reintegration Lines

We used the flyCRISPR website’s Optimal Target Finder tool (Gratz et al. 2014) to design chiRNAs utilizing PAM sites approximately 200 bp upstream of *Obp50a* and within 75 bp downstream of *Obp50d*. For each chiRNA we annealed complementary oligonucleotides with a *pU6-Bbs1-chiRNA* plasmid (Addgene catalog #45946) predigested with *Bbs1*. We constructed the homology-directed repair template by cloning approximately 1-kb up- and downstream of the *Obp50a–d* cluster into the up-and downstream *Sap1* and *Aar1* cloning sites, respectively, of the *pDsRed-attP* vector (Addgene catalog #51019). The two chiRNA plasmids, the *pBs-Hsp70-Cas9* plasmid (Addgene catalog # 46294), and the homology-directed repair template were injected into Canton S (B) embryos as a single mixture in a 2:2:5:10 ratio by molecular weight by Model System Injections (Durham, NC).

We constructed the *pattB-Obp50a–d-loxP-white^+^* vector by modifying the GenBank KC896839.1 *pattB* cloning vector using an In-Fusion kit (Takara Bio USA, Inc., Mountainview, CA) and designed primers with the company’s online tool (supplementary fig. S2, Supplementary Material online). We performed a series of site-directed mutagenesis reactions (fig. 1*B*) using a Q5 Site Directed Mutagenesis kit (New England Biolabs, Ipswich, MA) and designed primers with the NEBaseChanger online tool to generate eight plasmids containing different combinations of *Obp50a–d* constructs with intact genes or genes with premature termination codons or missense mutations (supplementary table S1, Supplementary Material online). The plasmids were injected into the F1 progeny of *Obp50[Δa–d; attP-loxP-DsRed-LoxP]* and PhiC31 integrase expressing flies by Model Systems Injections (fig. 1 and supplementary fig. S1, Supplementary Material online). Unless otherwise specified, enzymes used were from either New England Biolabs or Thermofisher Scientific (Waltham, MA). Primers are indicated in supplementary table S3, Supplementary Material online.

We confirmed proper orientation and location of the *attP-loxP-DsRed-LoxP* insert by Sanger sequencing PCR amplicons of the regions extending from >150 bp outside the homology arms to just inside the insertion. We confirmed orientation and location of the reinsertion lines by continuous Sanger sequencing of amplicons extending from upstream of the *attP* site to downstream of the *LoxP* sequence (fig. 1).

### RNA Sequencing

To prepare libraries for RNA sequencing we collected replicates of 30 mated flies, sexes separately, of up to two lines per genotype between 8:00 AM and 11:00 AM and flash froze them on dry ice in 2-ml tough microtubes (Thermofisher, Waltham, MA). Total RNA was extracted using a modified version of the RNAeasy plus mini kit (Qiagen, Hilden, Germany) protocol. Four 2.4-mm metal beads (Thermofisher) were added to each sample tube and the flies were homogenized in a bead mill (Thermofisher) for 2 min at 5 m/s, after which the RNA was eluted with a total of 30 μl H_2_O. We used the NuQuant +UDI, Drosophila AnyDeplete kit (Tecan, Männedorf, Switzerland) to deplete ribosomal RNA and prepare bar-coded cDNA libraries for sequencing after 17 cycles of amplification. We used the Qubit 1X HS dsDNA HS kit (Thermofisher) to quantify the libraries and high-sensitivity D1000 screentape (Agilent, Santa Clara, CA) to check the quality of size selection. We then normalized the libraries to 5 nM concentration and pooled them in order to get a final equimolar concentration of 3 nM. The pooled libraries were run on an S1 flow cell on the Illumina Novaseq6000 platform (Illumina, San Diego, CA).

### RNAseq Analysis

Raw reads were prepared for preprocessing by merging across lanes using a custom UNIX shell script. Adapter trimming, detection of abnormal polynucleotide sequences, filtering for low quality (*Q* < 20) and short (<35 nt) sequence reads and generation of basic sequence quality metrics were performed using the AfterQC pipeline (Chen et al. 2017). Detection of rRNA contamination was performed using the bbduk command from the BBTools package (Bushnell 2018) and consolidated rRNA sequences from the SILVA database (Quast et al. 2012). High-quality sequence reads were aligned to *Drosophila melanogaster* reference genome release 6 (version 6.13) using the allele-aware GSNAP aligner available within the GMAP package (Wu and Nacu 2010). The resulting SAM files were converted to BAM, sorted, and indexed using the samtools package (Li et al. 2009). Sorted and indexed BAM files were used for counting of meta-features (exons) using the featurecounts command within the Subread package (Liao et al. 2013). Uniquely mapped alignments were consolidated at the gene level and imported into R for subsequent analyses.

Genes with fewer than 25% nonzero read counts or a median count of <2 were excluded from further analyses. Filtered expression counts were normalized using GeTMM normalization (Smid et al. 2018). Two female samples, one *Obp50a^+^b^+^c^+^d^+^* and another *Obp50a^−^b^CD^c^+^d^−^*, were excluded from further analyses since they did not unambiguously group with the other female samples in a hierarchical clustering analysis. Differential expression was determined for each gene by specifying least squares means estimates for the transcriptional niche contrasts and conducting tests for epistasis within a linear mixed model ANOVA of the form $Y=\mathrm{Sex}+\mathrm{Genotype}+\mathrm{Sex}*\mathrm{Genotype}+\mathrm{Line}\left(\mathrm{Genotype}\right)+\;\varepsilon$, where Y is observed expression. Genes with Benjamini–Hochberg’s False Discovery Rate adjusted *P* value of less than 0.05 were considered statistically significant. Tests were performed with SAS version 9.4 (SAS Institute, Cary, NC).

Unsigned biweight midcorrelation networks were produced from filtered, normalized counts of the male samples of interest using the WGCNA R package (Zhang and Horvath 2005), with the maximum proportion of outliers constrained to be less than 10% as recommended by the WGCNA documentation. Soft thresholding powers were chosen to maximize the scale-free topology model fit and mean connectivity (see supplementary fig. S6, Supplementary Material online). Correlations between genes of interest were visualized with Cytoscape 3.7.2 (Shannon et al. 2003) using the yFiles Circular layout (yWORKS, Tübingen, Germany) after applying a hard threshold for correlation strength. All networks were constructed using the *Obp50a^+^b^+^c^+^d^+^* and *Obp50a^−^b^−^c^−^d^−^* data, whereas the networks of *Obp50a* (fig. 5*A*), *Obp50c* (fig. 5*B*), and *Obp50d* (fig. 5*C*) were also constructed from the *Obp50a^+^b^−^c^−^d^−^*, *Obp50a^−^b^−^c^+^d^−^*, and *Obp50a^−^b^−^c^−^d^+^* data, respectively. All networks in figure 6 and the *Obp50a–d* network (fig. 5*D*) were constructed from the *Obp50a^+^b^−^c^−^d^−^*, *Obp50a^−^b^+^c^−^d^−^*, *Obp50a^−^b^−^c^+^d^−^*, and *Obp50a^−^b^−^c^−^d^+^* data.

### Statistical Testing for Diversification and Epistasis

The same model used to test for overall significance of the Genotype term for a given phenotype was used to test for functional diversification between paralogs except that only samples of the *Obp50a^+^b^−^c^−^d^−^*, *Obp50a^−^b^+^c^−^d^−^*, *Obp50a^−^b^−^c^+^d^−^*, and *Obp50a^−^b^−^c^−^d^+^* genotypes were considered. A significant genotype term indicated diversification; otherwise, the paralogs were considered as having redundant effects. Epistasis was determined by the least squares means estimate $0=\left(\mathrm{Obp}50a^{+}b^{+}c^{+}d^{+}-\mathrm{Obp}50a^{-}b^{-}c^{-}d^{-}\right)\;-\left[\left(\mathrm{Obp}50a^{+}b^{-}c^{-}d^{-}-\mathrm{Obp}50a^{-}b^{-}c^{-}d^{-}\right)+\;\left(\mathrm{Obp}50a^{-}b^{+}c^{-}d^{-}-\mathrm{Obp}50a^{-}b^{-}c^{-}d^{-}\right)+\;\left(\mathrm{Obp}50a^{-}b^{-}c^{+}d^{-}-\mathrm{Obp}50a^{-}b^{-}c^{-}d^{-}\right)+\;\left(\mathrm{Obp}50a^{-}b^{-}c^{-}d^{+}-\mathrm{Obp}50a^{-}b^{-}c^{-}d^{-}\right)\right]+\varepsilon$. Significance was interpreted as the paralogs having epistatic effects; otherwise, they were considered additive. Epistatic effects with positive and negative estimates were deemed instances of “enhancing” and “suppressing” epistasis, respectively. Both the tests for epistasis and diversification were performed by sex when applicable.

### Starvation Stress Resistance

Flies were collected into vials containing 30–35 mated females and males, or 5–10 virgin females. The day before the assay, flies were separated into vials containing five flies of the same sex from each line. The next day, flies were transferred to vials containing 5 ml of starvation medium (1.5% agar in distilled H_2_O). The number of deceased flies was recorded ≥4 times per day (Harbison et al. 2004).

Statistical significance was determined by fitting a logistic distribution to the observed time of death using a parametric survival model of the form $Y=\mathrm{Genotype}+\mathrm{Line}\left(\mathrm{Genotype}\right)+\;\varepsilon$, where Y is observed time of death. Least squares means estimates were performed for contrasting transcriptional niches and Holm–Bonferroni-corrected *P* values were used for post hoc tests. Mated females, virgin females, and males were analyzed separately.

To test the hypothesis that increased fecundity correlates with starvation resistance, a separate assay was performed with mated females in which the vials used to hold the flies overnight were maintained and the emerging offspring counted. Correlation between the mean survival time versus the number of offspring produced per vial was assessed using a Standard Least Squares model.

### Larval Fat Content

To measure larval fat content, four males and four females were placed in vials on standard medium without yeast and allowed to lay eggs for 24 h. On day 5, 20% sucrose (w/v) in phosphate-buffered saline (PBS) was added to the vials and the floating wandering larvae were collected with a spatula and transferred to a 50-ml conical tube containing 10 ml of 10% sucrose in PBS. After gentle stirring, the larvae which floated to the top were counted after 2 min. The measurement was repeated after each incremental addition of 3 ml of 20% sucrose. When a total of 15 ml of 20% sucrose had been added, the number of larvae which remained at the bottom were also counted and added to the number of floating larvae to calculate the total number of larvae for each replicate and the percentage of floaters, which correlates with fat content (Hazegh and Reis 2016). Results were analyzed with a mixed model ANOVA according to supplementary table S3, Supplementary Material online.

### Sex Ratio

Vials with four males and four females were set up in food vials without added yeast (Day 0) and allowed to lay eggs for 3 days, after which they were discarded. Progeny were collected and sexed on days 9–15 (inclusive) as they emerged. Data were evaluated with a logistic regression model of the form $\mathrm{Sex}=\mathrm{Genotype}+\mathrm{Line}\left(\mathrm{Genotype}\right)+\mathrm{Vial}(\mathrm{Line},\;\mathrm{Genotype})+\;\varepsilon$. Least squares means estimates were performed and Holm–Bonferroni-corrected *P* values were used for post hoc tests. Correlation between the number of adult progeny per vial and percent female was assessed using a Standard Least Squares model. 

## Supplementary Material

Supplementary data are available at *Molecular Biology and Evolution* online.

## Supplementary Material

msab004_Supplementary_Data
