# Supplementary material for: Functional Diversification, Redundancy, and Epistasis among Paralogs of the Drosophila melanogaster Obp50a–d Gene Cluster
Source: Mol Biol Evol. 2021 Feb 9;38(5):2030–44. doi: 10.1093/molbev/msab004 (PMC8097280; doi:10.1093/molbev/msab004)
Supplement: msab004_Supplementary_Data [file msab004_supplementary_data.zip › Table S1.docx]

| **Table S1.** Primers Used | | |
| --- | --- | --- |
| **Purpose** | **Name** | **Sequence 5' -> 3'** |
| **CRISPR/*Cas9* chiRNA oligo (Fig. 1*AI*)** | chi_oligo_up_F | CTTCGTACCAATTGGGAGATCTCAA |
|  | chi_oligo_up_R | AAACTTGAGATCTCCCAATTGGTAC |
|  | chi_oligo_dn_F | CTTCGTCAGCTGAGCAAGACGTCTT |
|  | chi_oligo_dn_R | AAACAAGACGTCTTGCTCAGCTGAC |
| **CRISPR/*Cas9* repair template homology arm amplification (Fig. 1*AIb*)** | HA_amp_up_F | ttttCACCTGCttttTCGCgtgtacgggtggagcatctt |
|  | HA_amp_up_R | ttttCACCTGCttttCTACtctggggcttagtgcattttg |
|  | HA_amp_dn_F | ttttGCTCTTCtTATtgatggaatttATTGGGCTCAG |
|  | HA_amp_dn_R | tttGCTCTTCtGACgtatgcgtgatgccattgac |
| **CRISPR/*Cas9* repair template homology arm site-directed mutagenesis (Fig. 1*AIb*)** | HA_PAM_SDM_up_F | GATCTCAATGcTGTGCTAGGC |
|  | HA_PAM_SDM_up_R | TCCCAATTGGTAGCTATTTG |
|  | HA_PAM_SDM_dn_F | TTCTGGGTATgCGAAGACGTCTTGC |
|  | HA_PAM_SDM_dn_R | AAGCCCACTTGGCGGGAC |
| ***pattB-Obp50ad-loxP-w^+^* cloning (Fig. S2)** | Xba-LxP-Xba_F | TTTTTCTAGAataacttcgtataatgtatg |
|  | Xba-LxP-Xba_R | TTTTTCTAGAataacttcgtatagcataca |
|  | Xba-atB-Hin-Sap_F | TTTTTCTAGAcggtgcgggtgccagggcgtg |
|  | Xba-atB-Hin-Sap_R | aagcGGAAGAGCAAGCTTgtggagtacgcgcccgg |
|  | Hin-MCS-LxP-Hin_F | TTTTAAGCTTGGATCCGGGAATTGGGAATT |
|  | Hin-MCS-LxP-Hin_R | TTTTAAGCTTataacttcgtatagcataca |
|  | InFus_F | CCACAAGCTTGGATCTATCATTACTATAACTGGCATT |
|  | InFus_R | ACGAAGTTATTCTAGCTCAGTGATCAAAGCAGCT |
| **Site-directed mutagenesis reactions performed on the *pattB-Obp50ad-loxP-w^+^* vector to produce the eight reinsertion line genotypes (Fig. 1*B*)** | _50a_PTC_SDM_F | GAAAAATCAATCATTAAAGTATACGCCCTGTCCG |
|  | _50a_PTC_SDM_R | TAGGTTTAATAATTCCCTTTC |
|  | _50b_PTC_SDM_F | GGTGCTGCATTAATGAGGTTTCTTGTGGC |
|  | _50b_PTC_SDM_R | GAAGACATTTTGAAATTGCC |
|  | _50b_CD1_SDM_F | tgaatcTCTGTATAGGCACTGGGAC |
|  | _50b_CD1_SDM_R | tagcgggACTGCGAAAAGATTCTCTTTTAG |
|  | _50b_CD2_SDM_F | gcttcatgcacagtcTACCATGGTATACCTAACCG |
|  | _50b_CD2_SDM_R | atgggatatggcagagAGGTTGAGGAAGCCATGC |
|  | _50c_PTC_SDM_F | GCATATAGCCTAATGAATCTGCTCATTGC |
|  | _50c_PTC_SDM_R | CGGGCCATTTTTTTAGTC |
|  | _50d_PTC_SDM_F | CAAGCTGACTTAATGACTAATATTTATACCTG |
|  | _50d_PTC_SDM_R | TGAAGCATTTCGAGTTAC |
| **Confirmation of the presence of the *PhiC31 integrase* gene in fly crosses (Fig. S1*A*)** | Int_F | GCGGGGAAGAATCGATCAAG |
|  | Int_R | GCGTGCCTGATCTTGTTGAA |
| **Confirmation of the presence of the *Cre recombinase* gene in fly crosses (Fig. S1*B*)** | Cre_F | ctgttttgccgggtcagaaa |
|  | Cre_R | ccggtattgaaactccagcg |
| **PCR amplification of sequence between homology arms (Fig. 1)** | HA_up_F | GGAGGCTATCGAGTGCAATG |
|  | HA_dn_R | AGCCTGGATGAACTTGgtga |
| **PCR amplification of the transition between the homology arms and the adjacent genomic sequence (Fig. 1)** | HA_up_up_F | TGGCCTGAATTGACTTACTGG |
|  | HA_dn_dn_R | AAGTCGGCGAACTCCAAGTA |
|  | Red_up_R | GAGAACTCAAAGGTTACCCC |
|  | Red_dn_F | ATCAGCCATACCACATTTGT |
| **Sanger sequencing of the homology arms (Fig. 1)** | HA_seq_up_R | CAGTTGCCATTCCGACCATC |
|  | HA_seq_dn_F | CACAAAGTCCTGGAACGCAG |
| **Sanger sequencing of the *Obp50a-d* gene cluster (Fig. 1)** | _50ad_seq_F1 | GCTGGCCAAGGACATTTTT |
|  | _50ad_seq_F2 | AATGCATTTTGCATCGAGAA |
|  | _50ad_seq_F3 | ATGGGAGGATTGCAGAAATG |
|  | _50ad_seq_F4 | TGCACAAAGAAATTGGACGA |
|  | _50ad_seq_F5 | TCGCAGACAGGATTTCAATG |
|  | _50ad_seq_F6 | CAACCATTGTCCATCATCCA |
|  | _50ad_seq_F7 | TTTGGTCAATGGAGTCCACA |
