# Supplementary material for: Functional Diversification, Redundancy, and Epistasis among Paralogs of the Drosophila melanogaster Obp50a–d Gene Cluster
Source: Mol Biol Evol. 2021 Feb 9;38(5):2030–44. doi: 10.1093/molbev/msab004 (PMC8097280; doi:10.1093/molbev/msab004)
Supplement: msab004_Supplementary_Data [file msab004_supplementary_data.zip › Table S2.docx]

| **Table S2.** Mutations in Reinsertion Lines | | | | | |
| --- | --- | --- | --- | --- | --- |
|  |  | ***Obp50* gene** | | | |
| **Genotype** | **Plain Text Abbreviation** | ***-a*** | ***-b*** | ***-c*** | ***-d*** |
| ***Obp50a^+^b^-^c^-^d^-^*** | Obp50a | WT | L7X, L8X | L7X, L8X | W7X, V8X |
| ***Obp50a^-^b^+^c^-^d^-^*** | Obp50b | V7X, A8X | WT | L7X, L8X | W7X, V8X |
| ***Obp50a^-^b^CD^c^+^d^-^*** | Obp50cBCD | V7X, A8X | C68S, C72S, C148S, A152P, C158S | WT | W7X, V8X |
| ***Obp50a^-^b^-^c^+^d^-^*** | Obp50c | V7X, A8X | L7X, L8X | WT | W7X, V8X |
| ***Obp50a^-^b^-^c^-^d^+^*** | Obp50d | V7X, A8X | L7X, L8X | L7X, L8X | WT |
| ***Obp50a^+^b^+^c^+^d^+^*** | Obp50Pos | WT | WT | WT | WT |
| ***Obp50a^-^b^CD^c^-^d^-^*** | Obp50NegBCD | V7X, A8X | C68S, C72S, C148S, A152P, C158S | L7X, L8X | W7X, V8X |
| ***Obp50a^-^b^-^c^-^d^-^*** | Obp50Neg | V7X, A8X | L7X, L8X | L7X, L8X | W7X, V8X |

*WT = wild-type
