# Supplementary material for: Functional Diversification, Redundancy, and Epistasis among Paralogs of the Drosophila melanogaster Obp50a–d Gene Cluster
Source: Mol Biol Evol. 2021 Feb 9;38(5):2030–44. doi: 10.1093/molbev/msab004 (PMC8097280; doi:10.1093/molbev/msab004)
Supplement: msab004_Supplementary_Data [file msab004_supplementary_data.zip › Table S3.docx]

| **Table S3.** Summary of Mixed-Model ANOVA Tests | | | | | | |
| --- | --- | --- | --- | --- | --- | --- |
|  |  |  | **Fixed effects tests** | | | |
| **Phenotype** | **Fixed effect** | **Random effect** | **dfNum** | **dfDen** | **F ratio** | **Prob > F** |
| **Chill coma recovery** | Genotype | Date | 1 | 186 | 0.2192571 | 0.6402 |
|  | Sex |  | 1 | 186 | 9.6862057 | 0.0021 |
| **Startle response** | Genotype | Date | 1 | 394.3 | 0.0305033 | 0.8614 |
|  | Sex |  | 1 | 394.1 | 0.1292944 | 0.7194 |
| **Copulation latency** | Male genotype | Date | 1 | 173.9 | 0.0186845 | 0.8914 |
| **Copulation duration** | Male genotype | Date | 1 | 158.2 | 1.6312549 | 0.2034 |
| **Larval density** | Genotype | Rep[Genotype] | 7 | 148.0 | 0.936719 | 0.4801 |
|  | Vol. 20% sucrose |  | 1 | 779.0 | 5459.4664 | <.0001 |
