# Supplementary material for: Functional Diversification, Redundancy, and Epistasis among Paralogs of the Drosophila melanogaster Obp50a–d Gene Cluster
Source: Mol Biol Evol. 2021 Feb 9;38(5):2030–44. doi: 10.1093/molbev/msab004 (PMC8097280; doi:10.1093/molbev/msab004)
Supplement: msab004_Supplementary_Data [file msab004_supplementary_data.zip › Table S4.docx]

| **Table S4.** Genes membership in the redundant, additive, and *Obp50a-d* networks^a^ | | | | |
| --- | --- | --- | --- | --- |
| **Gene Symbol** | **FlyBase ID** | ***Obp50a-d* (fig. 5*D*)** | **Redundant (fig. 6*A*)** | **Additive (fig. 6*C*)** |
| *Acp54A1* | FBgn0083936 | 1^b^ | 1^b^ | 1^b^ |
| *Acp62F* | FBgn0020509 | 1 | 1 | 1 |
| *Anp* | FBgn0000094 | 1 | 1 | 1 |
| *CG10793* | FBgn0029656 | 1 | 0 | 1 |
| *CG10919* | FBgn0037514 | 1 | 1 | 1 |
| *CG11977* | FBgn0037650 | 1 | 1 | 1 |
| *CG12866* | FBgn0033955 | 1 | 1 | 1 |
| *CG13476* | FBgn0036441 | 1 | 1 | 1 |
| *CG15198* | FBgn0030283 | 1 | 1 | 1 |
| *CG15579* | FBgn0040906 | 1 | 1 | 1 |
| *CG17378* | FBgn0031858 | 1 | 1 | 1 |
| *CG2964* | FBgn0031462 | 1^b^ | 1 | 1^b^ |
| *CG31659* | FBgn0051659 | 1 | 1 | 1 |
| *CG31816* | FBgn0051816 | 1 | 1 | 1 |
| *CG32371* | FBgn0052371 | 1 | 0 | 1 |
| *CG34012* | FBgn0054012 | 1 | 1 | 1 |
| *CG42355* | FBgn0259701 | 1 | 1 | 0 |
| *CG42523* | FBgn0260428 | 1 | 1 | 1 |
| *CG42659* | FBgn0261531 | 1^b^ | 1^b^ | 1^b^ |
| *CG42798* | FBgn0261932 | 1 | 1 | 0 |
| *CG42827* | FBgn0262009 | 1 | 1 | 1 |
| *CG42847* | FBgn0262036 | 1 | 1 | 0 |
| *CG4306* | FBgn0036787 | 1 | 1 | 1 |
| *CG43185* | FBgn0262814 | 1 | 1 | 1 |
| *CG43209* | FBgn0262845 | 1 | 0 | 1 |
| *CG43288* | FBgn0262980 | 1 | 1 | 0 |
| *CG43319* | FBgn0263024 | 1 | 1 | 1 |
| *CG43350* | FBgn0263082 | 1 | 1 | 1 |
| *CG43668* | FBgn0263743 | 1 | 1 | 0 |
| *CG43675* | FBgn0263750 | 1 | 1 | 1 |
| *CG43829* | FBgn0264377 | 1 | 1 | 1 |
| *CG43861* | FBgn0264443 | 1 | 1 | 1 |
| *CG44139* | FBgn0264988 | 1 | 1 | 1 |
| *CG44198* | FBgn0265087 | 1 | 1 | 1 |
| *CG44574* | FBgn0265785 | 1 | 1 | 1 |
| *CG4669* | FBgn0035598 | 1 | 0 | 1 |
| *CG5024* | FBgn0039373 | 1 | 1 | 1 |
| *CG7094* | FBgn0032650 | 1 | 1 | 1 |
| *CG7768* | FBgn0036415 | 1^b^ | 1^b^ | 1^b^ |
| *CG9016* | FBgn0031751 | 1 | 1 | 1 |
| *CG9313* | FBgn0034566 | 1 | 1 | 1 |
| *CG9406* | FBgn0034592 | 1 | 1 | 1 |
| *CG9445* | FBgn0040674 | 1 | 1 | 1 |
| *CG9861* | FBgn0034844 | 1 | 1 | 1 |
| *CR31526* | FBgn0051526 | 1 | 1 | 0 |
| *CR42859* | FBgn0262106 | 1 | 1 | 1 |
| *CR43888* | FBgn0264480 | 1 | 1 | 1 |
| *CR43975* | FBgn0264706 | 1 | 1 | 0 |
| *CR44311* | FBgn0265370 | 1 | 0 | 1 |
| *CR44805* | FBgn0266040 | 1 | 1 | 1 |
| *CR44821* | FBgn0266068 | 1 | 1 | 1 |
| *CR45461* | FBgn0267017 | 1 | 1 | 1 |
| *Dup99B* | FBgn0250832 | 1^b^ | 1^b^ | 1^b^ |
| *goddard* | FBgn0036438 | 1 | 1 | 1 |
| *Gpo2* | FBgn0033190 | 1^b^ | 1^b^ | 1^b^ |
| *mil* | FBgn0267366 | 1 | 1 | 1 |
| *Mst84Dc* | FBgn0004174 | 1 | 0 | 1 |
| *Ntf-2r* | FBgn0032680 | 1 | 0 | 1 |
| *Obp22a* | FBgn0043539 | 1 | 1 | 1 |
| *Obp51a* | FBgn0043530 | 1 | 1 | 1 |
| *Rsph1* | FBgn0032478 | 1 | 1 | 1 |
| *Rsph4a* | FBgn0034957 | 1 | 0 | 1 |
| *Sfp23F* | FBgn0259949 | 1 | 1 | 1 |
| *Sfp26Ad* | FBgn0261055 | 1 | 1 | 1 |
| *Sfp33A2* | FBgn0259963 | 1 | 1 | 1 |
| *Sfp51E* | FBgn0259966 | 1 | 1 | 1 |
| *Sfp53D* | FBgn0259967 | 1^b^ | 1^b^ | 1^b^ |
| *Sfp96F* | FBgn0261061 | 1 | 1 | 1 |
| *Spn77Bc* | FBgn0036970 | 1 | 1 | 0 |
| *Tim13* | FBgn0036204 | 1 | 1 | 1 |
| ^a^1 indicates membership | | | | |
| ^b^Hub gene | | | | |
