# Supplementary figures and images for: Functional Diversification, Redundancy, and Epistasis among Paralogs of the Drosophila melanogaster Obp50a–d Gene Cluster
Source: Mol Biol Evol. 2021 Feb 9;38(5):2030–44. doi: 10.1093/molbev/msab004 (PMC8097280; doi:10.1093/molbev/msab004)

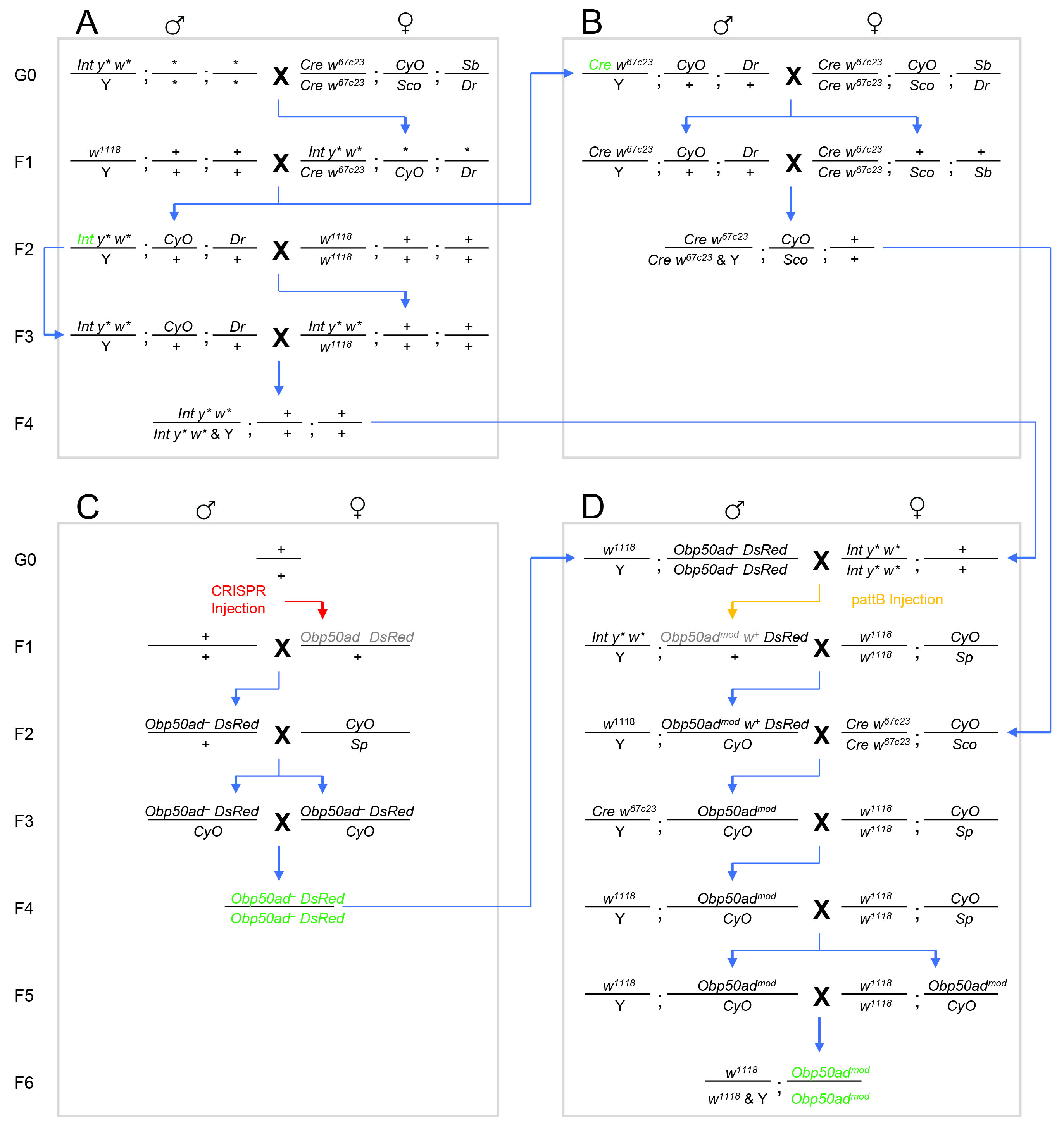

Supplement: msab004_Supplementary_Data [file msab004_supplementary_data.zip › Figure S1.jpg]

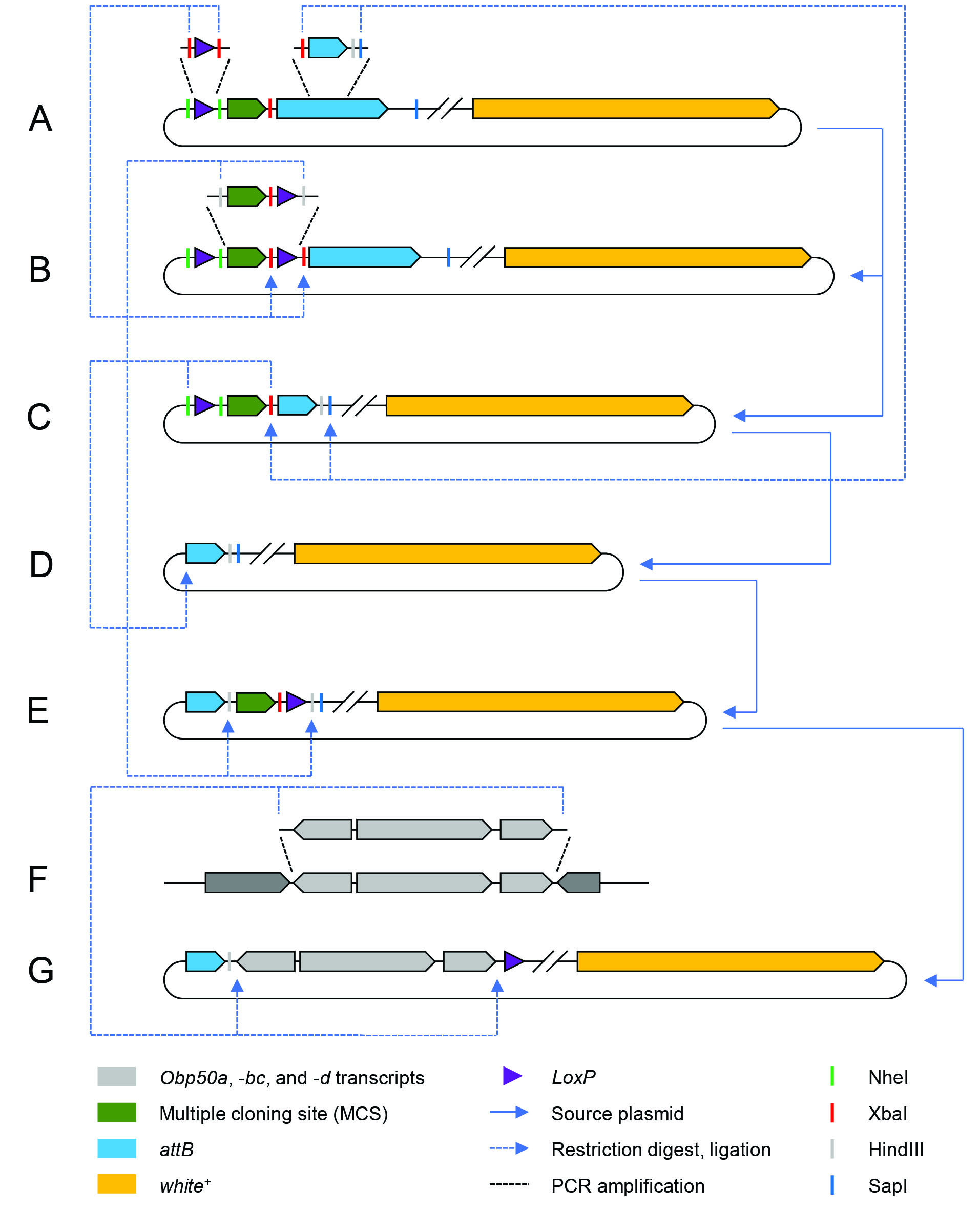

Supplement: msab004_Supplementary_Data [file msab004_supplementary_data.zip › Figure S2.jpg]

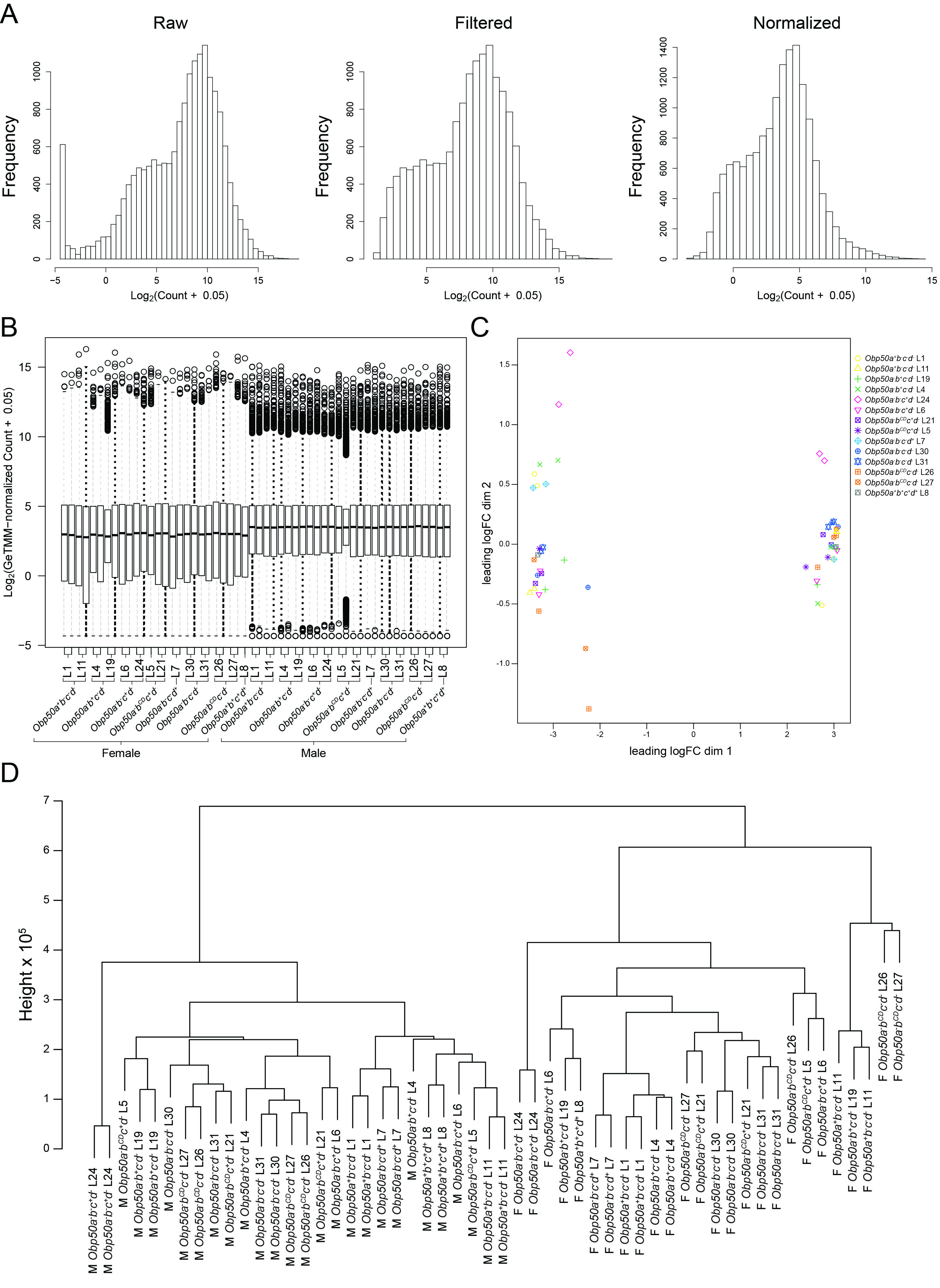

Supplement: msab004_Supplementary_Data [file msab004_supplementary_data.zip › Figure S3.jpg]

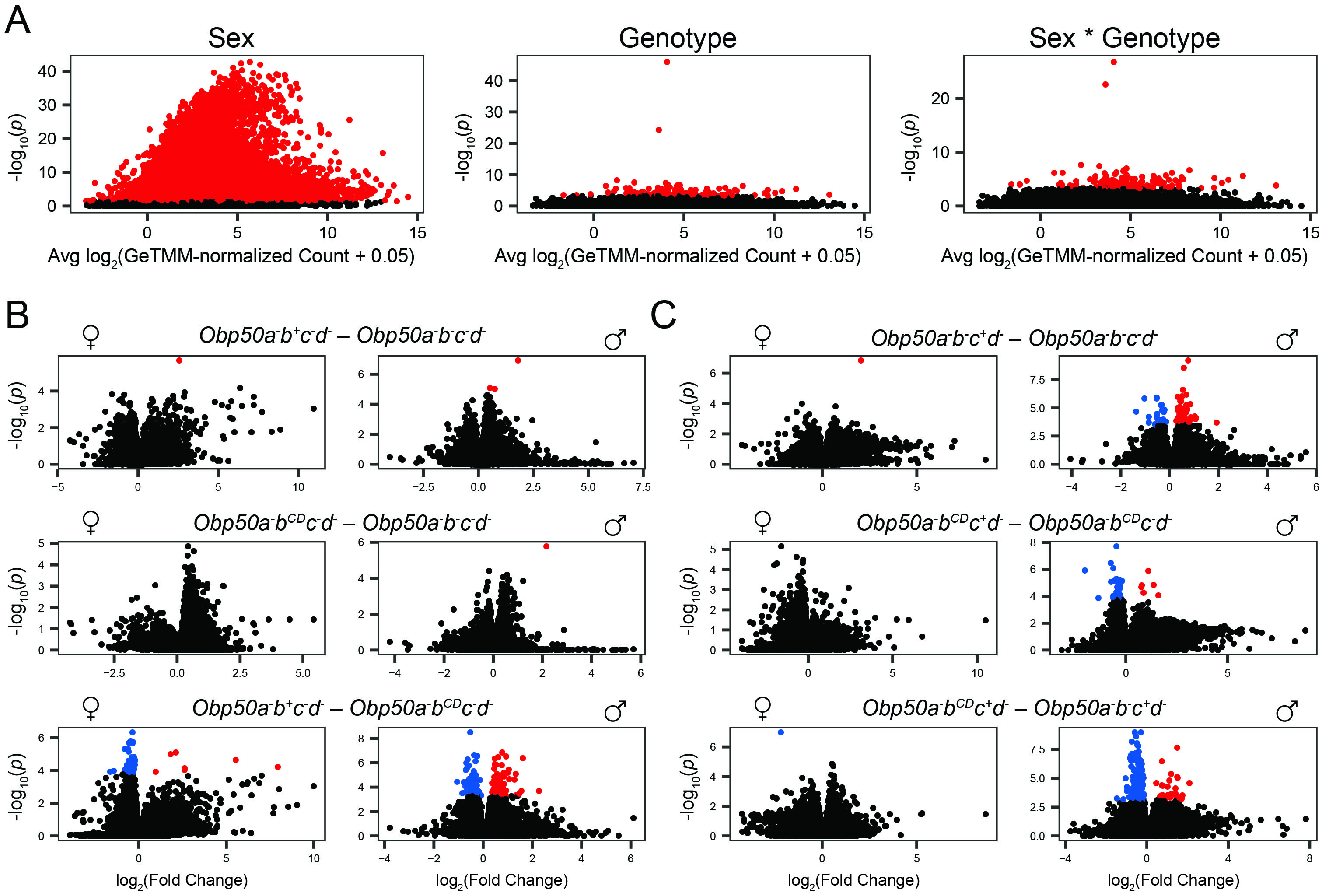

Supplement: msab004_Supplementary_Data [file msab004_supplementary_data.zip › Figure S4.jpg]

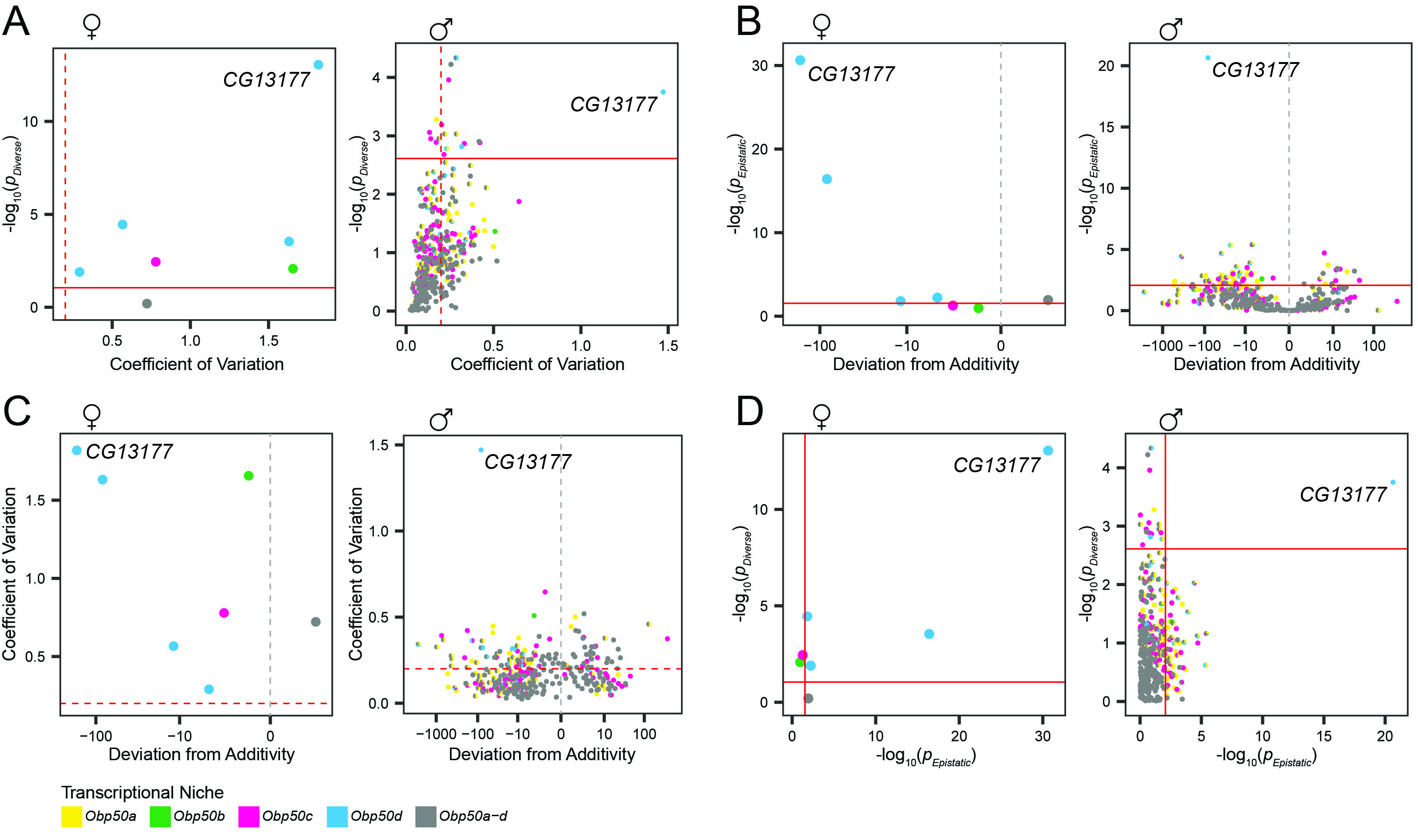

Supplement: msab004_Supplementary_Data [file msab004_supplementary_data.zip › Figure S5.jpg]

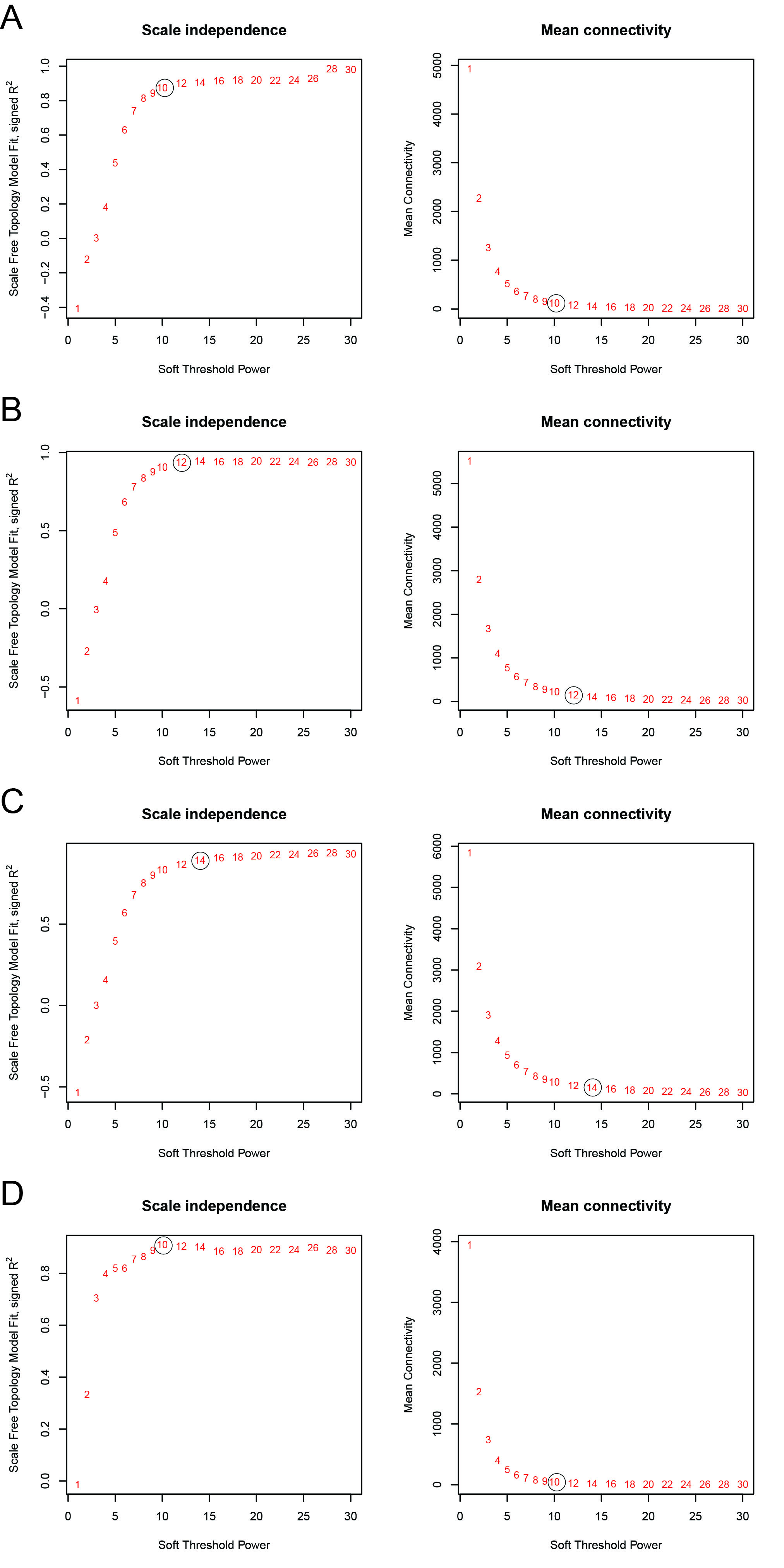

Supplement: msab004_Supplementary_Data [file msab004_supplementary_data.zip › Figure S6.jpg]
